# Vegetation drives the structure of active microbial communities on an acidogenic mine tailings deposit

**DOI:** 10.7717/peerj.10109

**Published:** 2020-10-21

**Authors:** Vanessa Gagnon, Michaël Rodrigue-Morin, Julien Tremblay, Jessica Wasserscheid, Julie Champagne, Jean-Philippe Bellenger, Charles W. Greer, Sébastien Roy

**Affiliations:** 1 Centre SÈVE, Département de biologie, Faculté des Sciences, Université de Sherbrooke, Sherbrooke, Québec, Canada; 2 National Research Council Canada, Energy, Mining and Environment, Montréal, Québec, Canada; 3 Centre SÈVE, Département de chimie, Faculté des Sciences, Université de Sherbrooke, Sherbrooke, Québec, Canada

**Keywords:** Mine tailings, Heavy metals, Primary succession, Vegetation density classes, PGPR microorganisms

## Abstract

Plant-microbe associations are increasingly recognized as an inextricable part of plant biology and biogeochemistry. Microbes play an essential role in the survival and development of plants, allowing them to thrive in diverse environments. The composition of the rhizosphere soil microbial communities is largely influenced by edaphic conditions and plant species. In order to decipher how environmental conditions on a mine site can influence the dynamics of microbial communities, we characterized the rhizosphere soil microbial communities associated with paper birch, speckled alder, and spruce that had naturally colonized an acidogenic mine tailings deposit containing heavy metals. The study site, which had been largely undisturbed for five decades, had highly variable vegetation density; with some areas remaining almost barren, and others having a few stands or large thickets of mature trees. Using Illumina sequencing and ordination analyses (redundancy analysis and principal coordinate analysis), our study showed that soil bacterial and fungal community structures correlated mainly with vegetation density, and plant species. Tailings without any vegetation were the most different in bacterial community structure, compared to all other areas on the mine site, as well as an adjacent natural forest (comparison plot). The bacterial genera *Acidiferrobacter* and *Leptospirillum* were more abundant in tailings without vegetation than in any of the other sites, while *Bradyrhizobium* sp. were more abundant in areas of the tailings deposit having higher vegetation density. *Frankia* sp. is equally represented in each of the vegetation densities and *Pseudomonas* sp. present a greater relative abundance in boreal forest. Furthermore, alder rhizosphere showed a greater relative abundance of *Bradyrhizobium* sp. (in comparison with birch and spruce) as well as *Haliangium* sp. (in comparison with birch). In contrast, fungal community structures were similar across the tailings deposit regardless of vegetation density, showing a greater relative abundance of *Hypocrea* sp. Tailings deposit fungal communities were distinct from those found in boreal forest soils. Alder rhizosphere had greater relative abundances of *Hypocrea* sp. and *Thelephora* sp., while birch rhizosphere were more often associated with *Mollisia* sp. Our results indicate that, with increasing vegetation density on the mine site, the bacterial communities associated with the individual deciduous or coniferous species studied were increasingly similar to the bacterial communities found in the adjacent forest. In order to properly assess and restore disturbed sites, it is important to characterize and understand the plant-microbe associations that occur since they likely improve plant fitness in these harsh environments.

## Introduction

In Québec, the first gold-mining operation began in the 1840s (Ministère de l’Énergie et des Ressources naturelles, 2018). At the time, regulations concerning mine site restoration did not exist, and once mining operations were completed, sites were generally abandoned. It is only recently that legislation concerning the obligation to restore mine sites has been put in place. In the meantime, 499 mining sites are considered abandoned in Québec (Ministère de l’Énergie et des Ressources naturelles, 2019). The abandoned sites have several common environmental problems, such as acidification and general soil degradation through the oxidation of sulfide minerals (such as iron pyrite (FeS_2_)) in the presence of water, oxygen and microorganisms and eventually through the production of acid mine drainage (AMD) (Kefeni, Msagati & Mamba, 2017; Simate & Ndlovu, 2014).

The nature of acid mine drainage (acidic pH and high metal(loid) concentration) alter the physical, chemical and biological structure of the ecosystem. This, combined with low organic matter content (top soil removed by mining activities), hinders plant colonization (Fellet et al., 2011; Wang et al., 2017). Moreover, the acidic pH that characterizes mine tailings, in addition to low plant density, result in low clay-humic complexes in these soils, leading to weak or almost no absorption, aggregation and sedimentation of metal(loid)s (Agbenyeku, Muzenda & Msibi, 2016; Akcil & Koldas, 2006; Pandey, Pandey & Misra, 2000). Thus, free metals in soils are able to migrate with runoff to streams and nearby forests unaffected by mining activities and this transfer of heavy metals into plants, water and the food chain can cause a public health problem (Ali, Khan & Sajad, 2013; Baalousha, Motelica-Heino & Le, 2006; Simate & Ndlovu, 2014).

Several remediation techniques including mechanical separation, pyrometallurgical separation, and chemical soil treatments are possible, but these techniques are costly in addition to being slow to implement (Mulligan, Yong & Gibbs, 2001). The use of plants to remedy mining sites is an increasingly popular measure, due to its low cost and ease of implementation. One such plant remedy is phytostabilization, which consists of reducing the mobility of soil contaminants using plants and their associated microorganisms (Ali, Khan & Sajad, 2013; Ma et al., 2011). In a mining context, the soils make the establishment of plants very difficult, since the plants are lacking in macroelements (i.e., C, N, P) essential for growth and they are confronted with constant oxidative stress caused by the excessive concentration of heavy metals (Sheoran, Sheoran & Poonia, 2010; Simate & Ndlovu, 2014). The critical period of establishment of a plant could be greatly facilitated by the inoculation of roots by microorganisms that can increase the adaptability and resilience of the plant and therefore enhance the success of phytostabilization (Grossnickel, 2005; Phieler, Voit & Kothe, 2013; Zhuang et al., 2007). Certain microorganisms in the rhizosphere, including PGPR bacteria (Plant Growth-Promoting Rhizobacteria) and mycorrhizal fungi, promote plant growth and allow the plant to adapt more easily to harsh environments (Bulgarelli et al., 2013; Sasse, Martinoia & Northen, 2018; Thijs et al., 2016). As an example, PGPR organisms can fix atmospheric nitrogen or solubilize phosphates, making these limiting macronutrients available to the plant. PGPR organisms could also secrete phytohormones (mostly auxins), that could enhance the germination and growth of the plant, and siderophores that promote metal mobility as they increase solubility (Berendsen, Pieterse & Bakker, 2012; Rajkumar et al., 2012).

Moreover, mycorrhizal fungi in association with plant roots extend their mycelial networks in the rhizosphere soil which leads to higher nutrient (i.e., P, N) and water capture than plant roots themselves (Jung et al., 2012). Indeed, Gamalero et al. (2009) reported that arbuscular mycorrhizal fungi could modify the root architecture of plants (depending on the host) thus resulting in more branching roots and eventually by increasing the nutrient uptake by the plant. Some arbuscular fungi (AM) and ectomycorrhizal (ECM) fungi act as buffers between the soil and the plant roots which could decrease heavy metal bioavailability by chelating them in their vacuoles, improving plant resistance to heavy metal stress in soil (Colpaert et al., 2011; Khan, 2005; Miransari, 2010; Thijs et al., 2016).

The mine site studied was characterized as being naturally colonized by trees, shrubs, and grasses. The vegetation density was quite variable on site, from a lack of plants to dense groves composed of several species (Tardif et al., 2019). The mine tailings had a pH ranging from 3 to 9, and arsenic concentrations ranging from 1 to 1951 ppm. The objectives of this study were to determine the main environmental parameters that structured the microbial communities in soil and the rhizosphere, and begin to decipher how environmental conditions on a mine site can influence the dynamics of microbial communities, which are a key factor in plant survival and development in the natural environment. Understanding how plant-microbial associations developed in a mining context could help develop tailored microbial inocula to prepare seedlings used in mine site restoration and facilitate their establishment.

## Materials & Methods

### Study area

**Figure 1:**
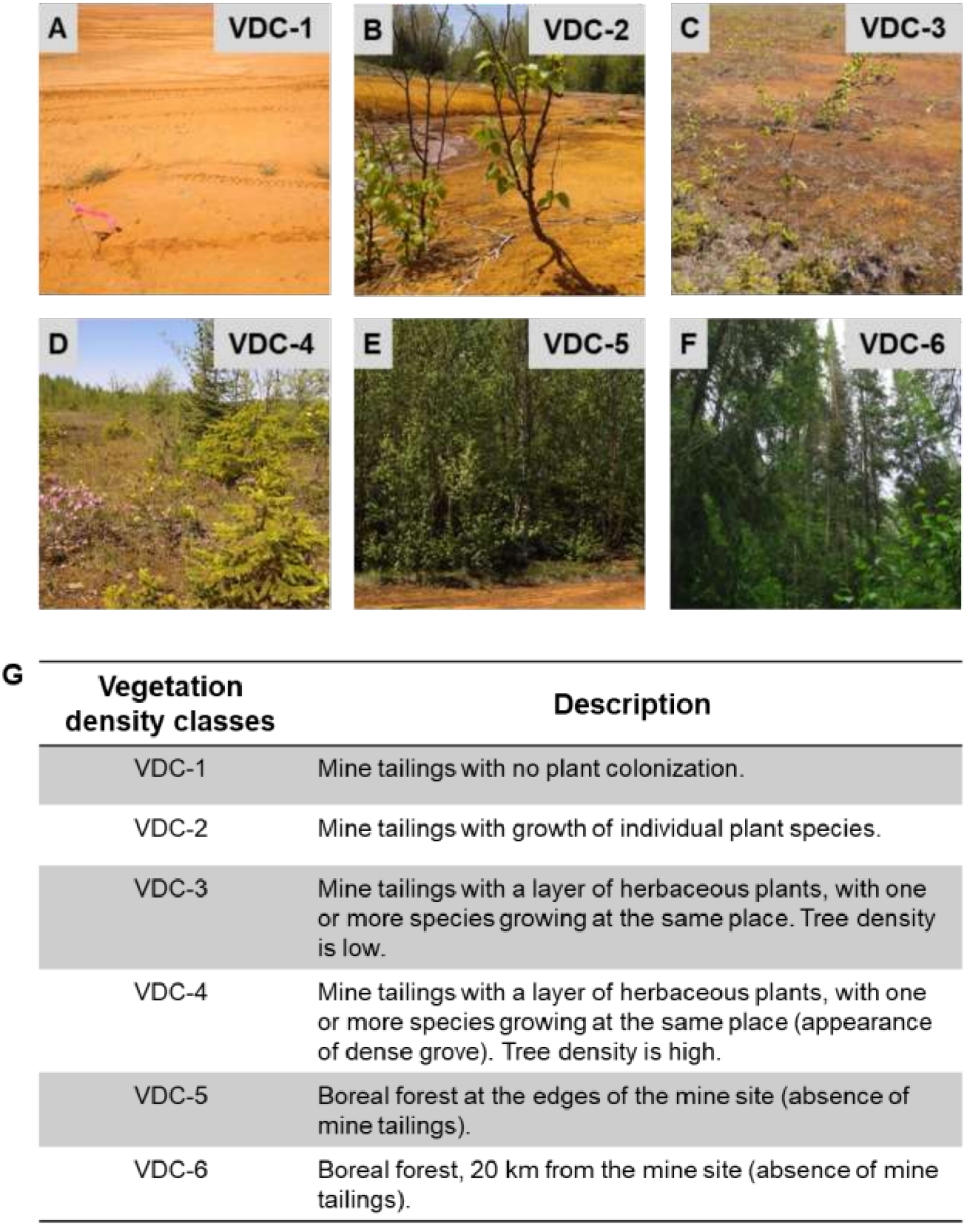
Vegetation density classes (VDC) and description. (A) VDC-1. (B) VDC-2. (C) VDC-3. (D) VDC-4. (E) VDC-5. (F) VDC 6. (G) Description of the different vegetation density classes.

The studied mine site is located in Abitibi-Témiscamingue, Québec (Canada), at the Southern limit of the boreal forest belonging to the bioclimatic domain *Abies balsamea-Betula papyrifera.* The forest is mainly composed of balsam fir (*Abies balsamea*), black spruce (*Picea marinara*), white spruce (*Picea glauca*), paper birch (*Betula papyrifera*) and Jack pine (*Pinus banksiana*) (Bergeron et al., 2004; Kneeshaw & Bergeron, 1998). The mine site is home to several plant species, which grow naturally on tailings and mainly include alder (*Alnus incana* ssp. *rugosa*), paper birch and spruce (*Picea* sp.) (Gagnon et al., 2020; Tardif et al., 2019). For this study, the mine site was organized into four classes constituting the vegetation density classes (VDC): VDC-1, VDC-2, VDC-3, and VDC-4 (Fig. 1). For comparison, two adjacent boreal forest areas were studied: VDC-5, which was near the mine site and VDC-6 at 20 km from the mine site.

### Bulk soil and rhizosphere soil sampling

Sampling was conducted at the beginning of September 2015 and 2016. The number of sample replicates for plants (for rhizosphere and bulk soil) is detailed in Table S1. In VDC-2, 3 and 4, trees of each species (alder, paper birch and spruce) were chosen according to their availability and size, selecting primarily only small plants (approximately 0.5 m in height) (Table S1). These provided plant samples, and soil/tailings samples. To collect fifty gram soils/tailings samples in areas without plants (VDC-1), in the forest adjacent to the mine site (VDC-5) and in the distant far boreal forest (VDC-6), samples were randomly collected under the litter (if present) in the first 30 cm of soil. More specifically, bulk soil (and not rhizosphere soil) was collected in VDC-1, 5 and 6, due to the lack of plants (VDC-1) or the difficulty in VDC-5 and VDC-6 of harvesting the rhizosphere soil of mature trees in the forest. All plant and soil/tailings samples were kept at 4 °C until they arrived at the laboratory. Then, the roots were shaken in a beaker to collect the rhizosphere soil (approximately 20 g). The rhizosphere and bulk soil were frozen at −20 °C until RNA extraction.

### Soil physico-chemical analysis

Soil pH, water content and elemental analyses by inductively coupled plasma mass spectrometry (ICP-MS) (except for total nitrogen and organic content) were performed previously (Gagnon et al., 2020). For elemental analysis, samples were digested using nitric acid (HNO_3_). For plant tissues, this digestion resulted in complete mineralization of the samples. Digestion of soil samples with this strong acid (HNO_3_) is an established method known to extract all metals that can become environmentally available, but does not extract metal included in the mineral fraction of recalcitrant minerals (e.g., silicates) (Osher et al., 2006; Tuovinen et al., 2015, U.S. EPA, 1996; Zhi Dang, Liu & Haigh, 2002). Digestions were carried out on a heating plate (DigiPREP Mini, SCP SCIENCE, Québec, Canada). Briefly, samples were crushed using a mortar and pestle in liquid nitrogen. Twenty-five mg of powder were transferred to 15 ml digiTUBES^®^ (SCP SCIENCE) to which 1 ml of concentrated nitric acid (HNO_3_ Optima™, trace metal grade, Fisherbrand, Fair Lawn, NJ, USA) was added. Tubes were heated at 45 °C for 30 min. Then, another 1 ml of nitric acid was added, and the tubes were heated at 65 °C for 2 h. Digestates were brought to 10 ml with milliQ^®^ water (Millipore, Québec, Canada). Elemental quantitation was performed using a Thermo Fisher XSERIES 2 ICP-MS (Thermo Fisher Scientific, Bremen, Germany) and PlasmaLab software (Thermo Electron Corp. 2004. version 2.6.1.335. [PlasmaLab]. Germany: PC). Rhodium was used as an internal standard. The SLR5 certified water standard was used as an internal control to calibrate the ICP-MS. The studied metals were: sodium (^23^Na), magnesium (^24^Mg), aluminum (^27^Al), phosphorus (^31^P), potassium (^39^K), calcium (^44^Ca), titanium (^47^Ti), vanadium (^51^V), chromium (^52^Cr), manganese (^55^Mn), iron (^57^Fe), cobalt (^59^Co), nickel (^60^Ni), copper (^65^Cu), zinc (^66^Zn), arsenic (^75^As), selenium (^82^Se), molybdenum (^95^Mo), silver (^107^Ag), cadmium (^111^Cd), antimony (^121^Sb), barium (^137^Ba), tungsten (^182^W), thallium (^205^Tl) and lead (^206^Pb). Total nitrogen was determined by the combustion method (AOAC 990.03) and organic content was quantified by the calcination method (AGDEX 522, method PR-2) in an external laboratory (Groupe EnvironeX, https://www.labenvironex.com/en/). The results are presented in Table S2.

### RNA extraction, library preparation and sequencing

Total RNA was extracted from environmental samples and then separated from DNA. RNA-based analysis was preferred over DNA-based analysis, as it provides data on microbial populations that are present and active, and not simply present (DNA).

Two grams of frozen bulk soil or rhizosphere was weighed into tubes containing 1.5 g of silica beads. Total RNA was extracted, purified and eluted as described in the RNA PowerSoil^®^ Total RNA Isolation kit (MO BIO Laboratories Inc., #12866-25, CA, USA). For removal of residual DNA, the RNA extracts were subjected to DNase treatment using the DNase I kit (Thermo Fisher Scientific, #18068015, Canada). Efficiency of DNA removal was verified through universal PCR amplification of the bacterial 16S rRNA genes and visualization of PCR product on 2% agarose gels to confirm the absence of DNA.

The 16S rRNA gene sequences (eubacteria and archaea) and the fungal nuclear ribosomal internal transcribed spacer (ITS) region were first amplified by RT-PCR using the OneStep RT-PCR kit (Qiagen, #220211, Canada). The specific primer sequences (Integrated DNA Technologies, Canada) for these regions (16S rRNA gene and ITS) are described in Table S3. These were chosen to identify bacterial and fungal classes and genera, as reported in the current literature (Kaiser et al., 2016; Sun et al., 2017; Urbanovà, Šnajdr & Baldrian, 2015; Žifčáková et al., 2016). For each region (16S rRNA gene and ITS), four pairs of primers with additional bases inserted between the sequencing primer overhang and the gene specific sequence (staggered pad) were used proportionally among the samples to create diversity in the sequence reads, thereby improving the quality of sequencing data. For amplification of the 16S rRNA region, a peptide nucleic acid (PNA) clamp was used to prevent amplification of eukaryotic RNA. PNA chloroplast and PNA mitochondrial blockers were used as proposed in Hong, Yang & Zhuang (2016).

Amplicons were visualized on a 2% agarose gel. The samples were purified using AMPure XP magnetic beads (Beckman Coulter Genomics, #A63881, CA, USA), and a second short amplification of 8 cycles was carried out with the Nextera XT index kit (Illumina^®^ #FC-131-1001, set A, B, C and D, CA, USA) to insert different index primers on the amplicons, according to the protocol in the Illumina “16S Metagenomic Sequencing Library Preparation” guide (Part #15044223 Rev. B). Unique codes were added to each sample by amplifying 5 µl of the purified PCR product with 25 µl of KAPA HIFI HotStart Ready Mix, 300 nM each Nextera XT Index Primer (Illumina^®^ Inc., CA, USA) and 10 µl UltraPure™ DNase/RNase-Free Distilled Water for a total volume of 50 µl. Thermal cycling conditions were as follows: 3 min at 98 °C, 8 cycles of 30 s at 98  °C, 30 s at 55 °C, 30 s at 72  °C, and a final elongation step of 5 min at 72  °C. Indexed amplicons were purified with magnetic beads as described above. Amplicon products were quantified using the Quant-it PicoGreen™ dsDNA Assay Kit according to the manufacturer’s instruction (Thermo Fisher Scientific, MA, USA) and combined in an equimolar ratio. The amplicon pool was verified with a Bioanalyzer 2100 (Agilent Technologies, CA, USA) to confirm the size of the amplicons and to visualize the potential presence of primer dimers or adapters, in which case an additional purification step of the amplicon pools with Select SPRI (Beckman Coulter, #B23319, CA, USA) beads was performed. Finally, the pool was denatured with 0.2 N NaOH, and the internal control PhiX was added at 5% (Illumina^®^ Inc., #FC-110-3001, CA, USA). The samples were sequenced on the MiSeq instrument (Illumina^®^ Inc., #SY-410-1003, CA, USA) using the MiSeq v2 500 cycle kit (Illumina^®^ Inc., #MS-102-2003, CA, USA) at the National Research Council Canada in Montréal, Québec (Canada).

### Bioinformatics analysis

Sequencing results were analyzed using a previously described methodology (Tremblay et al., 2015; Yergeau et al., 2015). Briefly, both the 16S rRNA gene and ITS amplicons were separately filtered, assembled, trimmed and controlled for quality. Sequencing data was analyzed using AmpliconTagger (Tremblay & Yergeau, 2019). Raw reads were scanned for sequencing adapters and PhiX spike-in sequences and remaining reads were merged using their common overlapping part with FLASH (Magoč & Salzberg, 2011). Primer sequences were removed from merged sequences and remaining sequences were filtered for quality such that sequences having an average quality (Phred) score lower than 30 or one or more undefined bases (N) or more than 5 bases lower than a quality score 15 were discarded.

The generation of OTU tables was done with a three round clustering strategy. Quality controlled sequences were dereplicated at 100% identity. These 100% identity clusters were denoised at 99% identity using dnaclust v3 (Ghodsi, Liu & Pop, 2011). Clusters having an abundance of 3 or more reads were scanned for chimeras with UCHIME denovo and UCHIME reference-based algorithms, using the Broad Institute 16S rRNA gene Gold reference (http://microbiomeutil.sourceforge.net). The remaining clusters were clustered at 97% identity (dnaclust) to generate OTUs.

The taxonomic assignment of bacterial and fungal OTU results was performed with the RDP classifier (Bayesian classifier) with a training model constructed from the Greengenes database (version 13.5) (DeSantis et al., 2006; Wang et al., 2007). Fungal OTUs were classified using a training model constructed with the United Database (Kõljalg et al., 2013).

The RDP classifier gives a score (0 to 1) to each taxonomic depth of each OTU. Each taxonomic depth having a score ≥ 0.5 was kept to reconstruct the final lineage. Taxonomic lineages were combined with the cluster abundance matrix obtained above to generate a raw OTU table. From that raw OTU table, an OTU table containing bacteria only was generated. This latter OTU table was normalized using the edgeR R packages (Robinson, McCarthy & Smyth, 2010) to obtain a Counts Per Million (CPM) normalized OTU table. A multiple sequence alignment was then obtained by aligning OTU sequences on a Greengenes core reference alignment (DeSantis et al., 2006) using the PyNAST v1.2.2 aligner (Caporaso et al., 2010). Alignments were filtered to keep only the hypervariable region of the alignment. Alpha (observed species (Chao1 index)) and beta (Bray Curtis distances) diversity metrics and taxonomic summaries were then computed using the QIIME v1.9.1 software suite (Caporaso et al., 2010; Kuczynski et al., 2011) using the normalized OTU table.

Sequencing data are available on the NCBI Sequence Read Archive (SRA) portal under accession number PRJNA634113.

### Statistical analysis

A matrix of environmental properties needed for the redundancy analysis (RDA) and the MANOVA was performed as described in Gagnon et al. (2020). Briefly, the species data matrix (y) was transformed by a centered log ratio since OTU data are considered compositional data (Macklaim, 2014). Since there were 174 bacterial classes and 1906 bacterial genera and 31 fungal classes and 889 fungal genera, for uniformity we chose to keep the 30 most abundant bacterial and fungal classes and genera for RDA. The RDA was performed using the “rda” function with the significant explanatory variables selected by the “ordiR2step” function. A second RDA was performed on the significant explanatory variables. The significance of the explanatory variable (red arrows) was verified using the “anova.cca” function (Table S4). The RDA are presented in type II scaling to allow a better interpretation of the correlation between matrices (x) and (y) (Legendre & Legendre, 2009).

Principal coordinate analysis (PCoA) was performed on normalized OTU tables. A distance matrix was created via the “vegdist” function, and PCoA was performed using the “pcoa” function. MANOVA was conducted to determine if significant differences in active bacterial populations and active fungal populations were present across VDC, or between plant species (terHorst, Lennon & Lau, 2014; Liu et al., 2015b; Wu et al., 2017; Warton & Hudson, 2004). Table 1 presents the *p*-values indicating the significance of differences observed between the active microbial populations between the various VDCs, and between plant species.

**Table 1:** *P*-values from MANOVA (nperm = 999) performed between the VDC and plant species’ soils and rhizospheres presented in Fig. 3. Bacterial (A) and fungal (B) populations between vegetation density classes (VDC). Bacterial (C) and fungal (D) populations between plant species (p_s).

VDC	1	2	3	4	5
**(A)**					
2	0.002	–	–	–	–
3	0.002	0.007	–	–	–
4	0.002	0.960	0.007	–	–
5	0.002	0.002	0.792	0.002	–
6	0.002	0.649	0.165	0.544	0.007
**(B)**					
2	0.467	–	–	–	–
3	0.109	0.012	–	–	–
4	0.022	0.002	0.792	–	–
5	0.002	0.002	0.003	0.002	–
6	0.002	0.002	0.002	0.002	0.038

p_s	*Alnus rugosa*	*Betula papyrifera*
**(C)**		
*Betula papyrifera*	0.170	–
*Picea* sp.	0.330	0.330
**(D)**		
*Betula papyrifera*	0.560	–
*Picea* sp.	0.830	0.900

The microbial community profiles of the most abundant bacterial and fungal classes and genera were determined using the Shiny software (In-house R script) that allows the illustration of 20 classes.

To characterize the degree of change, and possible trends in microbial diversity across VDC, Chao1 boxplots were performed (GraphPad Prism 8 software for OS X, v8.1.2). We used the “Identify outlier” (method: ROUT, Q = 1%) function in GraphPad Prism to remove outliers from Chao1 datasets.

Statistical differences between vegetation density classes and plant species were analyzed in R using the “Kruskal.test”, “Dunn.test”, “lm” and “Anova” functions. Significant differences in bacterial richness between VDC and plant species were investigated using non-parametric Kruskal–Wallis followed by Dunn’s multicomparison test as the dataset did not meet the normality requirement after transformation. Significant differences in fungal richness between VDC were investigated using unbalanced ANOVA (sum of squares of type III) analysis followed by Tukey’s post-hoc test.

To determine what microbial populations were shared (vs unique) amongst VDC or plant species, the following online software was used: http://bioinformatics.psb.ugent.be/webtools/Venn/. The software sorted our metadata and OTU table (at the genus level), producing a list of most to least abundant populations. The 100 most abundant bacterial OTUs, and 100 most abundant fungal OTUs were then categorized into Venn tables that list which OTUs were shared or unique amongst VDC or plant species (Tables S5 and S6).

**Figure 2:**
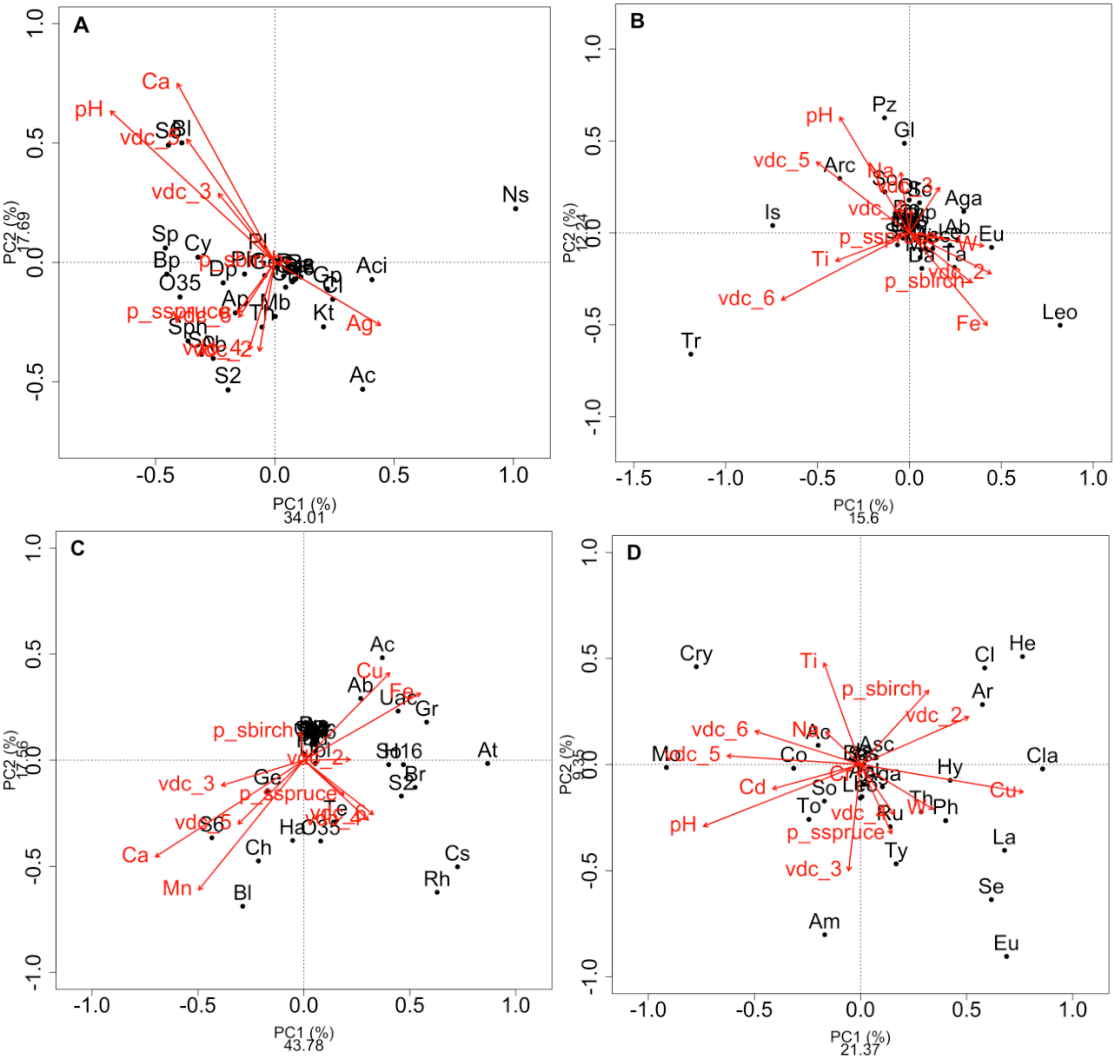
Redundancy analysis (RDA) of the effect of soil environmental variables. Redundancy analysis (RDA) of the effect of soil environmental variables on the class composition of bacteria (A) and fungi (B) and genus composition of bacteria (C) and fungi (D) in soils and rhizosphere. Red arrows indicate soil edaphic parameters that significantly explained the 20 most abundant bacterial or fungal classes or genera (black dots). The lowercase letters in parentheses represent the maximum depth of taxonomy (g: genus, f: family, c: class, o: order, p: phylum, k: kingdom). p_s: plant species, VDC: vegetation density classes. (A): Ab: Actinobacteria (c), Ac: Acidobacteria (c), Aci: Acidimicrobia (c), Ap: Alphaproteobacteria (c), B: Bacteria (k), Bp: Betaproteobacteria (c), Bl: Blastocatellia (c), Caz: Candidatus azambacteria (c), Ch: Chlamydiae (c), Cl: Clostridia Cy: Cytophagia (c), Dp: Deltaproteobacteria (c), Gp: Gammaproteobacteria (c), Ge: Gemmatimonadaceae (c), Kt: Ktedonobacteria (c), Mb: Melainabacteria (c), Ns: Nitrospira (c), O35: OPB35 soil group (c), Pa: Parcubacteria (p), Ph: Phycisphaetea (c), Pl: Planctomycetaciae (c), P: Proteobacteria (p), S2: Acidobacteriaceae_subgroup 2 (c), S6: Acidobacteriaceae_subgroup 6 (c), Sa: Saccaribacteria (p), Sph: Sphingobacteria (c), So: Solibacteres (c), Sp: Spartobacteria (c), Th: Thermoleophilia (c), TM6: TM6 (p); (B): Ab: Agaricostilbomycetes (c), Aga: Agaricomycetes (c), Arc: Archaeorhizomycetes (c), Art: Arthoniomycetes (c), Asc: Ascomycota (p), Bas: Basidiomycota (p), Bl: Blastocladiomycetes (c), Ch: Chytridiomycetes (c), Chp: Chytridiomycota (p), Cy: Cystobasidiomycetes (c), Da: Dacrymycetes (c), Do: Dothiodeomycetes (c), Eu: Eurotiomycetes (c), Ex: Exobasidiomycetes (c), F: Fungi (k), Gl: Glomeromycetes (c), Is: *Incertae sedis* (c), Le: Lecanoromycetes (c), Leo: Leotiomycetes (c), Mi: Microbotyomycetes (c), Mo: Monoblepharidomycetes (c), Or: Orbiliomycetes (c), Pu: Pucciniomycetes (c), Pz: Pezizomycetes (c), Sa: Saccharomycetes (c), Sc: Schizosaccharomycetes (c), So: Sordariomycetes (c), Ta: Taphrinomycetes (c), Tr: Tremellomycetes (c), Us: Ustilaginomycetes (c); (C): 03: 0319-6G20 (f), Ac: *Acidobacteraceae* (c), Ab: *Acidibacter* (g), Aq: *Aquicella* (g), At: *Acidothermus* (g), B: Bacteria (k), Bd: *Bdellovibrio* (g), Bl: Blrii4 (f), Br: *Bryobacter* (g), Caz: Candidatus azambacteria (c), Cs: *Candidatus solibacter* (c), Ch: *Chthniobacter* (g), Ge: *Gemmata* (g), Gr: *Granulicella* (g), H16: H16 (g), Ha: *Haliangium* (g), Le: *Legionella* (g), O35: OPB35 soil group (c), Pa: Parcubacteria (p), P: Proteobacteria (p), UAc: Uncultured-*Acidobacteraceae* (c), UPl: Uncultured-*Planctomycetaciae* (c), Rh: *Rhizomicrobium* (g), S2: *Acidobactericaceae* _subgroup 2 (c), S6: *Acidobactericaceae*_subgroup 6 (c), Sa: Saccharibacteria (p), SM: SM2D12 (f), So: *Sorangium* (g), Te: Tepidisphaeraceae (c), TM6: TM6 (p); (D): Ac:* Archaeorhizomyces* (g), Aga: *Agarocimycetes* (c), Agl: *Agaricales* (o), Am: *Amphinema* (g), Ar: *Articulospora* (g), Asc: *Ascomycota* (p), Bas: Basidiomycota (p), Cl: *Cladophialospora* (g), Cla: *Claussenomyces* (g), Co: *Cortinarius* (g), Cry: *Cryptococcus* (g), Eu: *Eurotiomycetes* (c), F: Fungi (k), He: *Helotiales* (o), Hy: *Hyaloscypha* (g), In: *Inocybe* (g), Is: *Incertea sedis* (c), La: *Lactarius* (g), Leo: Leotiomycetes (c), Mol: *Mollisia* (g), Mo: *Mortierella* (g), Rh: *Rhizoscyphus* (g), Ru: *Russula* (g), Sc: *Schizangiella* (g), Se: *Serendipita* (g), So: *Sordariomycetes* (c), Th: *Thelephora* (g), To: *Tomentella* (g), Ty: *Tylospora* (g).

## Results

**Figure 3:**
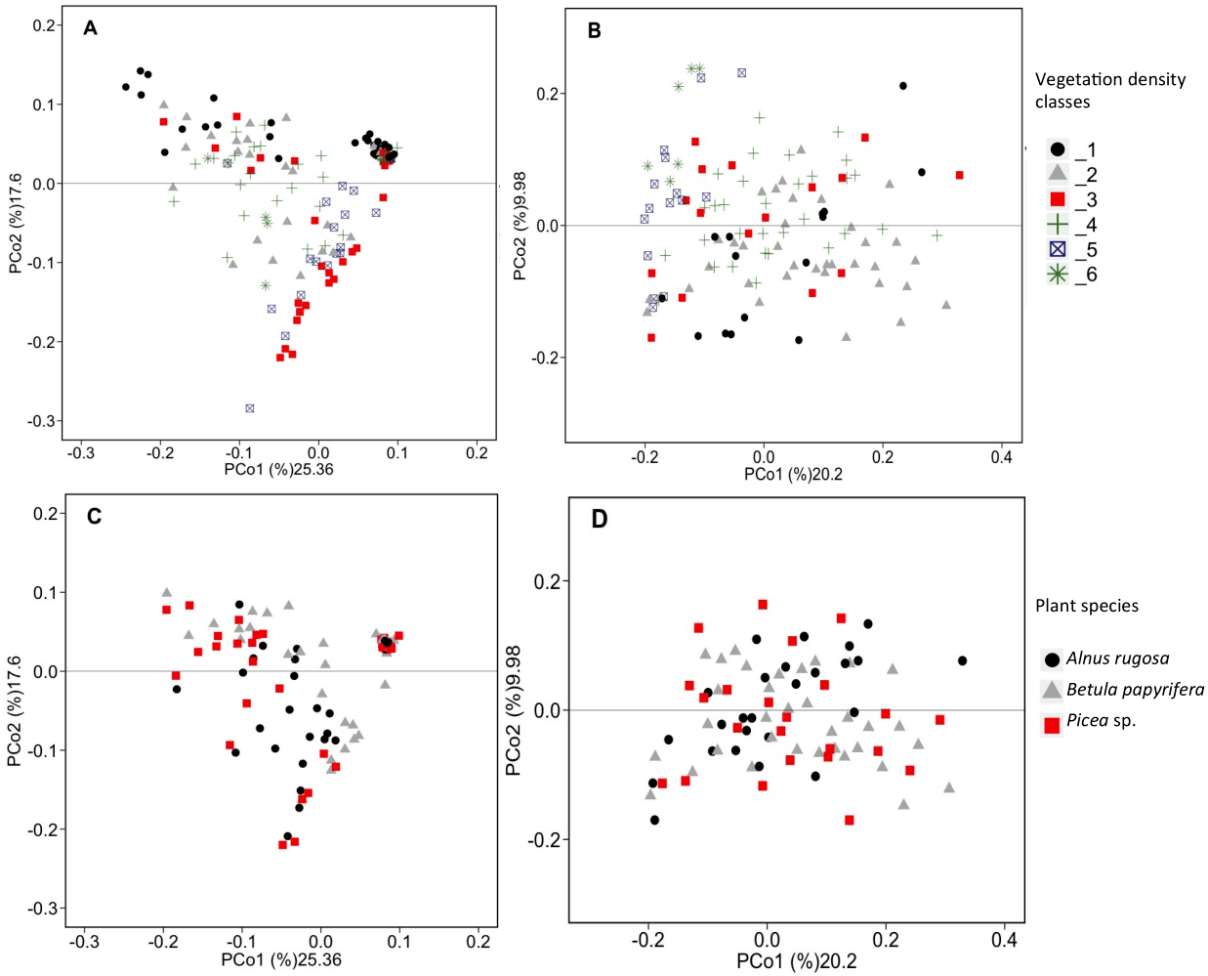
PCoA analysis of bacterial and fungal total functional population. PCoA analysis of bacterial (A, C) and fungal (B, D) total functional population between different vegetation density classes (A, B) and plant species (C, D) in soils and rhizosphere.

RDAs allowed us to highlight which environmental parameters (such as VDC, plant species, pH, water content and metal concentration) drove bacterial and fungal communities on the mine site (VDC-1 to 4) and boreal forest (VDC-5 and 6). The 30 most abundant classes and genera represented 84.99% and 38.35% of the total bacterial population (at the class and genus levels) and 99.14% and 79.72% of the total fungal population (at the class and genus levels) in tailings and soils. The first two axes of the RDA explained 51.85%, 27.84%, 61.34% and 32.72% of the variance in bacterial classes, fungal classes, bacterial genera and fungal genera, respectively (Fig. 2). Each of the RDA significant axes (red arrows) were tested for levels of significance (*p*-values are shown in Table S4). The VDC and p_s (plant species) were the most significant axes on each of the RDAs shown in Fig. 2 (Figs. 2A, 2B, 2C and 2D: (VDC *p*-value = 0.001 in each graphic, p_s *p*-value = 0.008, 0.001, 0.007 and 0.001 respectively). There were other significant axes (e.g., pH, Ca, Cd, Cr, Cu, Fe, Na, Mn, Ti, W) on Fig. 2, but VDC and p_s best explained the structure of bacterial and fungal communities. Further analysis (PCoA) revealed that most differences in bacterial communities were noted in VDC-1 compared to other VDC (Fig. 3A and Table 1A). Total bacterial communities also differed between alder and birch rhizospheres (Fig. 3C and Table 1C). Differences in fungal total communities were found in VDC-5 and 6, compared to all other VDC in tailings (VDC-1, 2, 3 and 4) (Fig. 3B and Table 1B). In addition, differences were noted between alder and spruce rhizosphere fungal communities (Fig. 3D and Table 1D).

**Figure 4:**
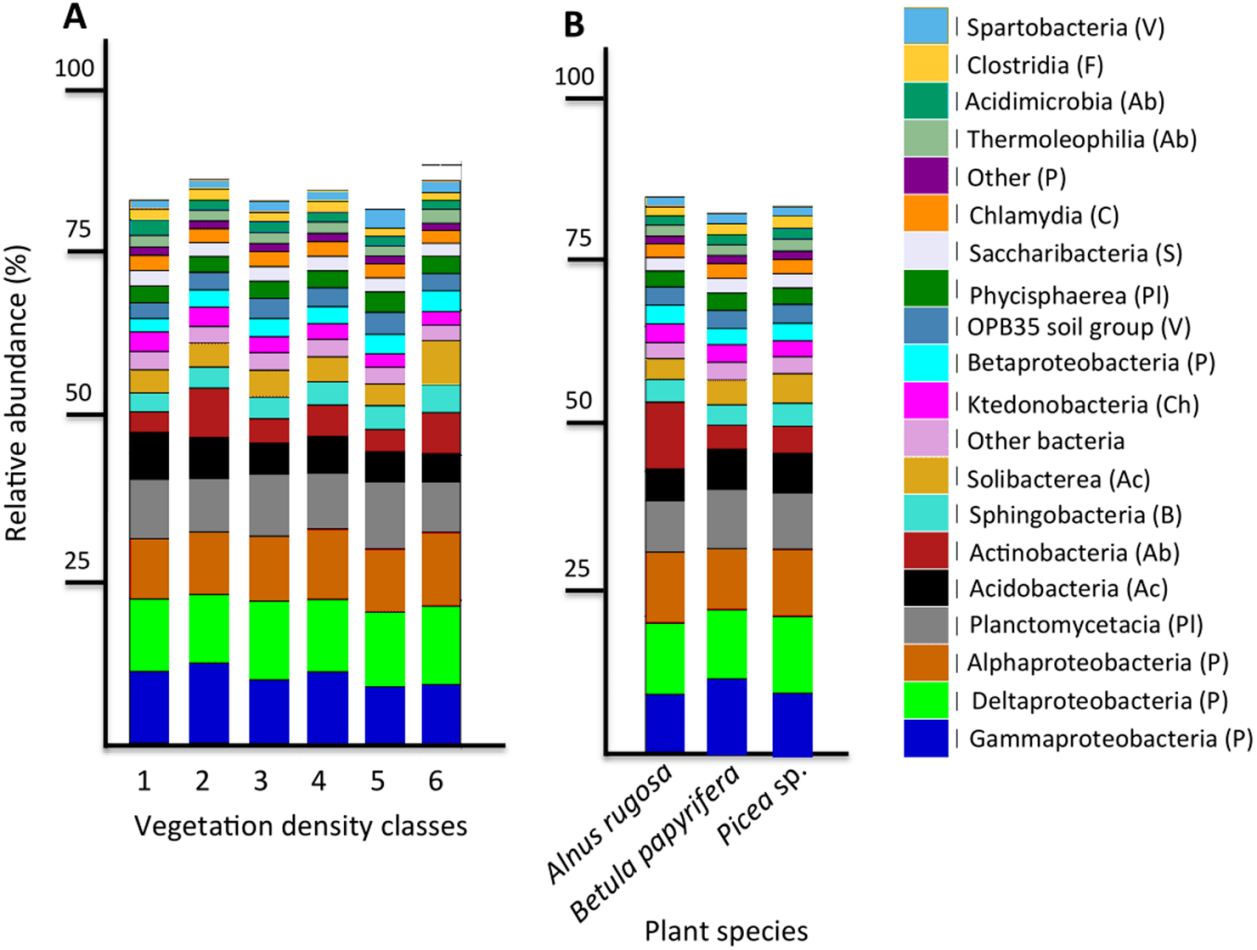
Bacterial community profile (relative abundance of transcripts) of the 20 most abundant classes in soils and the rhizospheres of different vegetation density classes (A) and plant species (B). Letters in parentheses indicate phyla (Ac: Acidobacteria, Ab: Actinobacteria, B: Bacteroidetes, C: Chlamydiae, Ch: Chloroflexi, F: Firmicutes, P: Proteobacteria, Pl: Planctomycetes, S: Saccharibacteria, V: Verrucomicrobia). Other bacteria designates bacteria that were not identified to phylum level.

**Figure 5:**
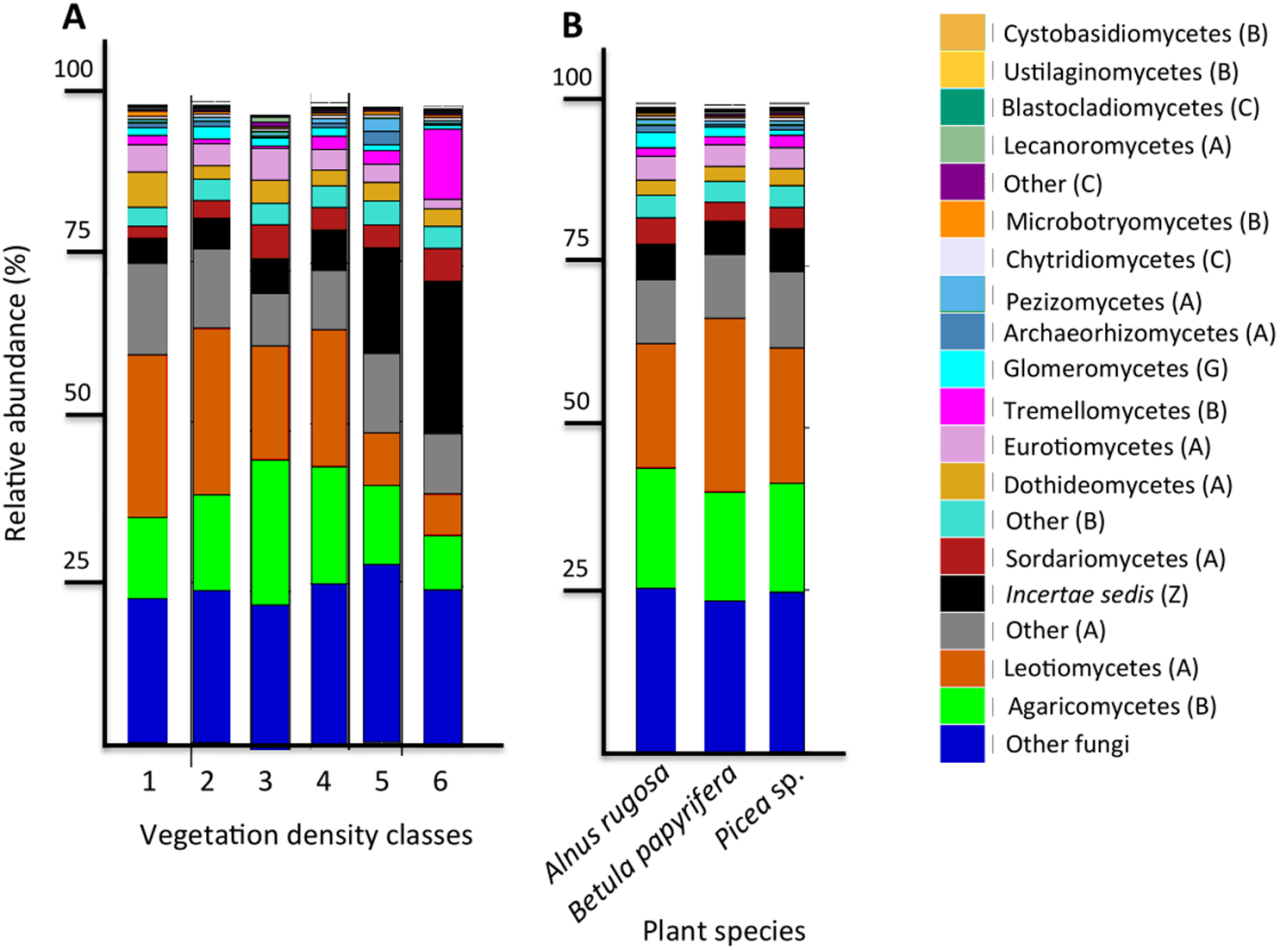
Fungal community profile (relative abundance of transcripts) of the 20 most abundant taxa in soils and the rhizospheres of different vegetation density classes (A) and plant species (B). Letter in parentheses represents phyla (A: Ascomycota, B: Basidiomycota, C: Chytridiomycota, G: Glomeromycota, Z: Zygomycota). Other fungi designates fungi that were not identified to phylum level.

The six most abundant classes of bacteria for each VDC and p_s were Gammaproteobacteria (7.9%–11.5%), Deltaproteobacteria (9.5%–10.8%), Alphaproteobacteria (8.3%–10.4%), Planctomycetacia (7.0%–9.3%), Acidobacteria (3.9%–6.4%) and Actinobacteria (2.8%–5.7%) (Fig. 4 and Table S7 A). For fungi, the classes “others (with no phylum)”, Agaricomycetes (11.3%–26.3%), Leotiomycetes (5.7%–18.7%), “others (Ascomycota phyla)” (9.3%–14.5%), *Incertae sedis* (3.6%–19.7%) and Sordariomycetes (2.3%–8.3%) were the six most abundant (Fig. 5 and Table S7B). The 100 most abundant bacterial and fungal genera in each of the VDC as well as p_s are listed in Tables S5 and S6.

**Figure 6:**
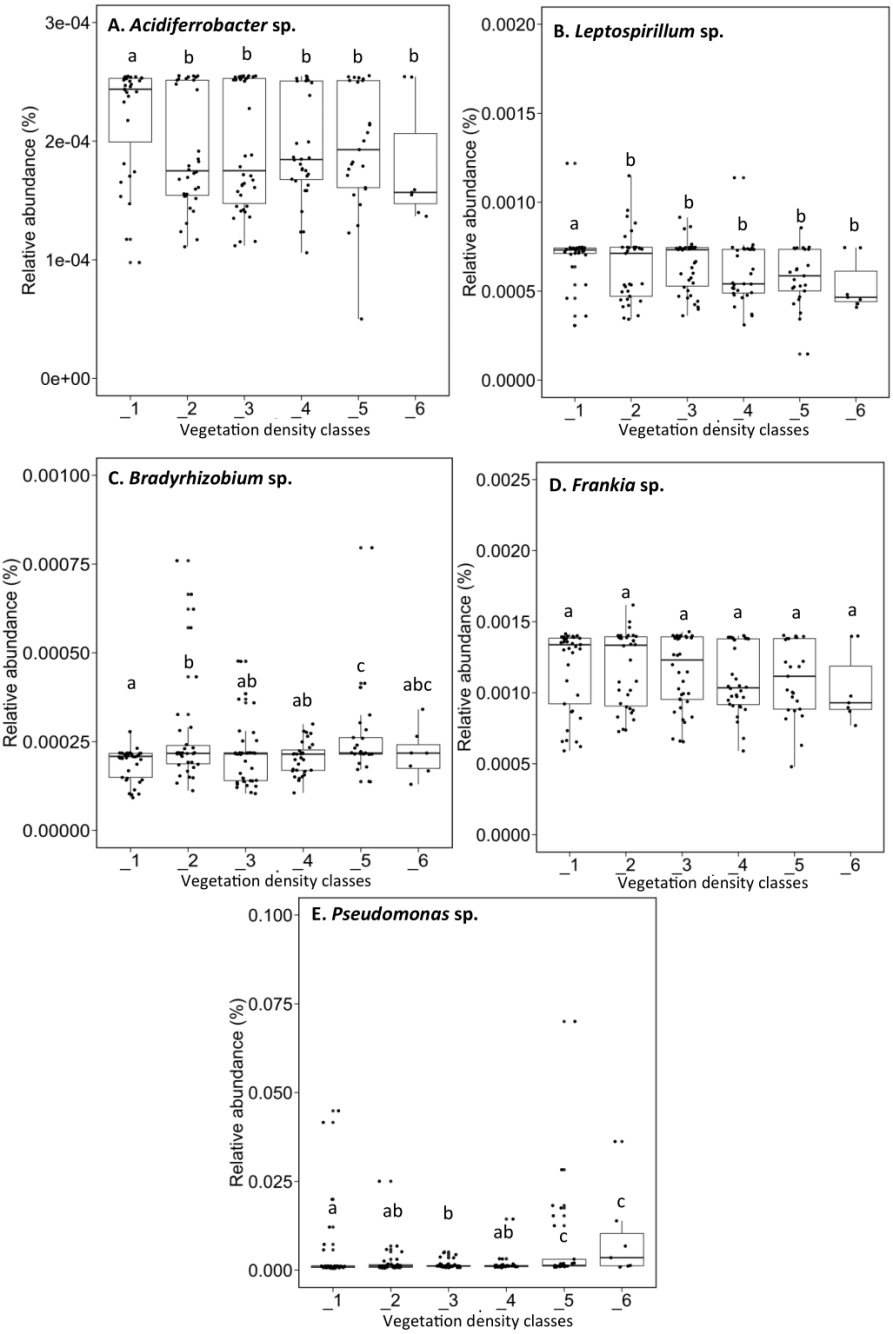
Relative abundance (%) of various bacterial species present in soils and the rhizosphere in the different vegetation density classes. (A) *Acidiferrobacter sp.*, (B) *Leptospirillum sp.*, (C) *Bradyrhizobium sp.*, (D) *Frankia sp.*, (E) *Pseudomonas sp.*. The different lowercase letters represent a significant difference after Kuskal–Wallis followed by Dunn’s multicomparison test.

*Acidiferrobacter* sp. (median abundance: 0.0002%) and *Leptospirillum* sp. (median abundance: 0.0007%) had a greater relative abundance in VDC-1 (Figs. 6A and 6B). *Bradyrhizobium* showed a greater relative abundance in the rhizosphere on the vegetated tailings and the boreal forest (median abundances: 0.000216% to 0.000218%) (VDC-2 to 6) (Fig. 6C). *Frankia* sp. showed an equal relative abundance (median abundances: 0.0009%–0.0013%) in each of the vegetation densities (Fig. 6D). *Pseudomonas* sp. showed the highest relative abundance in the boreal forest (VDC-5 and 6) (median abundances: 0.0013% and 0.0035%, respectively) in comparison to all other VDC (Fig. 6E) (median abundances: 0.001158% to 0.001163%).

**Figure 7:**
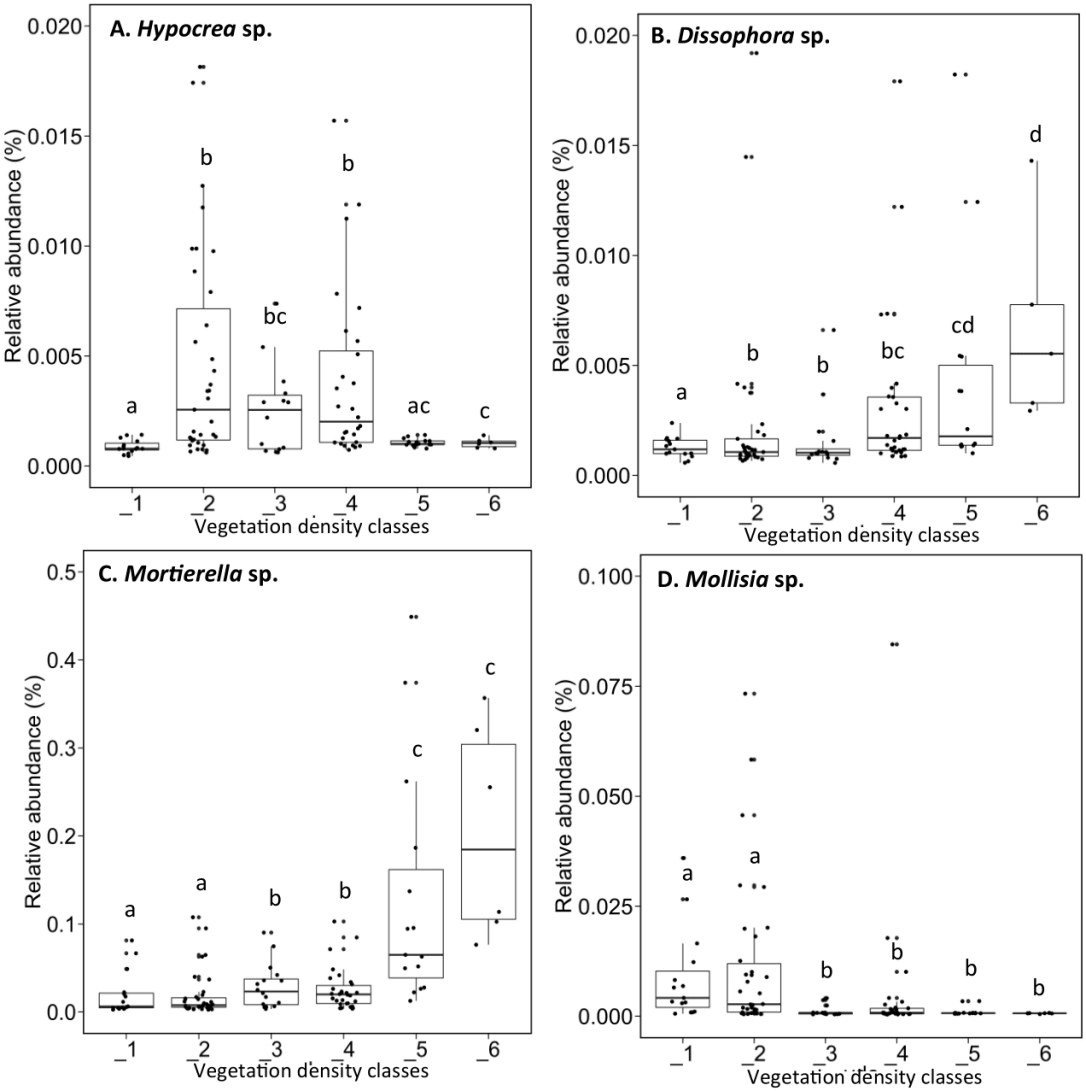
Relative abundance of transcripts (%) of various fungi species present in soils and the rhizospheres in the different vegetation density classes. (A) *Hypocrea sp.*, (B) *Dissophora sp.*, (C) *Mortierella sp.*, (D) *Molissia sp.*. The different lowercase letters represent a significant difference after Kruskal–Wallis followed by Dunn’s multicomparison test.

The fungal genus *Hypocrea* had higher relative abundances (median abundances: 0.0024% to 0.0029%) in tailings with low to high vegetation density (VDC-2, 3 and 4) compared to barren tailings of VDC-1 (Fig. 7A). In the presence of no or low vegetation (VDC-1 and 2) *Mollisia* showed a greater relative abundance (median abundances: 0.0028% and 0.0041%, respectively) compared to other VDC (Fig. 7D) (median abundances: 0.0007% to 0.0008%). *Dissophora* sp., and *Mortierella* sp. showed the highest relative abundance in VDC 5 and 6 (Figs. 7B and 7C) (*Dissophora* sp. median abundances: 0.0021% and 0.0066%, *Mortierella* sp. median abundances: 0.0648% and 0.1846%). Other fungal genus tended to be found in higher abundance in forest soils (VDC-5 and/or VDC-6) compared to tailings: *Cortinarius* sp. (VDC-5, median abundance 0.0042%), *Neonectria* sp. (VDC-5 and 6, median abundances 0.0003% and 0.0331%, respectively) and *Rhinocladiella* sp. (VDC-5, median abundance 0.0002%). VDC-1 to VDC-4 median abundances for these genus were 0.0017% to 0.0019% for *Cortinarius* sp., 0.0002% for *Neonectria* sp. and 0.0001% for *Rhinocladiella* sp.

**Figure 8:**
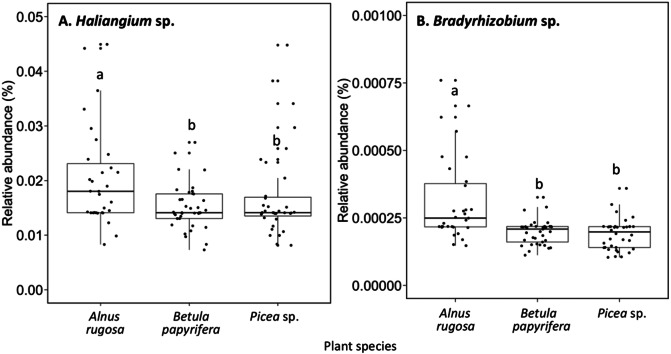
Relative abundance of transcripts (%) of various bacterial species present in the rhizosphere of different plant species. (A) *Haliangium sp.*, (B) *Bradyrhizobium sp.*. The different llowercase letters represent a significant difference after Kruskal–Wallis followed by Dunn’s multicomparison test.

**Figure 9:**
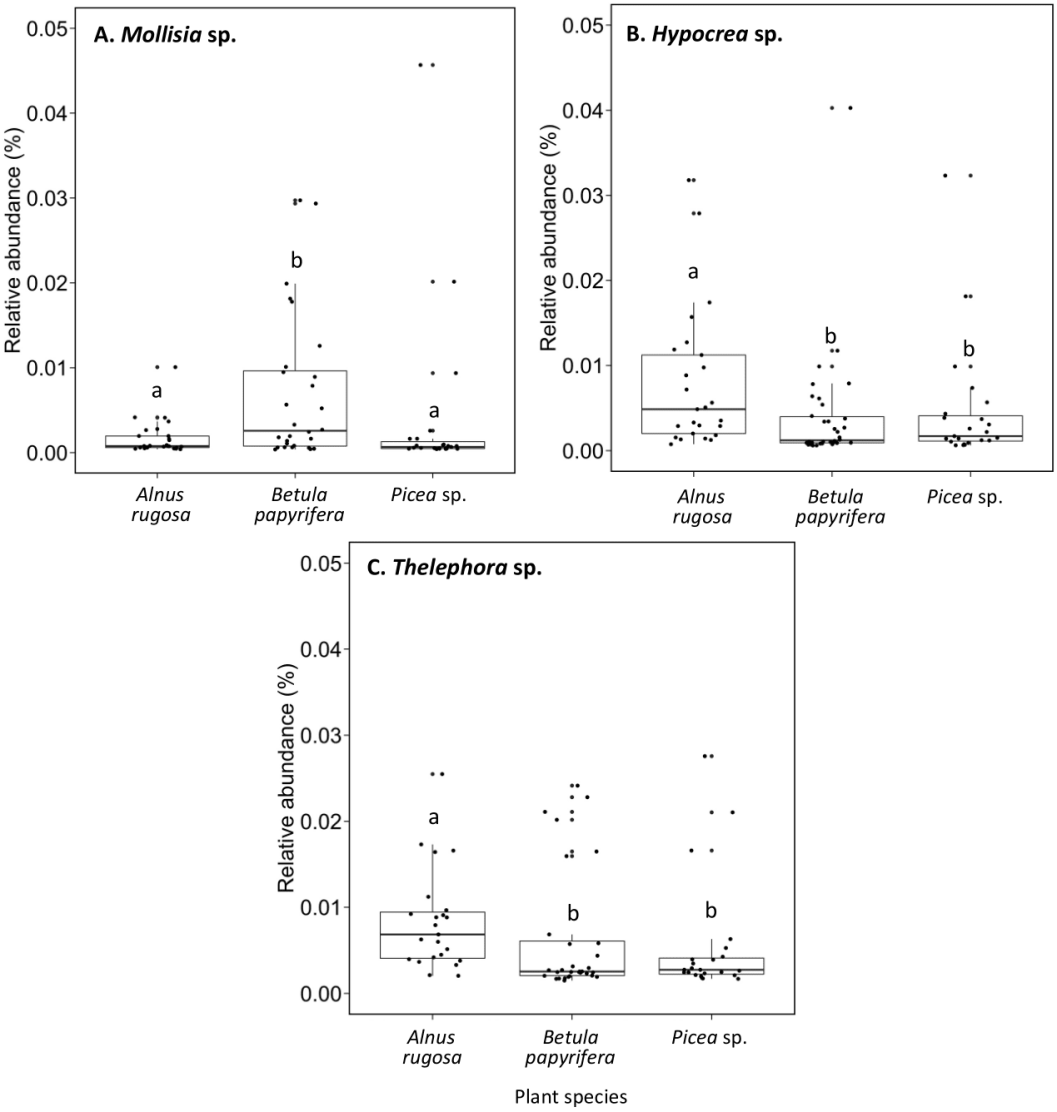
Relative abundance of transcripts (%) of various fungi species present in the rhizosphere of different plant species. (A) *Mollisia sp.*, (B) *Hypocrea sp.*, (C) *Thelephora sp.*. The different llowercase letters represent a significant difference after Kruskal–Wallis followed by Dunn’s multicomparison test.

The structure of bacterial communities differed significantly between alder and birch rhizospheres (Fig. 3C and Table 1C). *Haliangium* were relatively more abundant in alder rhizosphere (median abundance: 0.0194%), compared to that of birch and spruce (median abundances: 0.0149% and 0.0141%, respectively) (Fig. 8A). *Bradyrhizobium* were also more abundant in alder rhizosphere (median abundance: 0.0003%), compared to that of birch and spruce (median abundances: 0.00019% and 0.00022%, respectively) (Fig. 8B). Fungal community structures were also significantly different between alder and spruce rhizospheres (Fig. 3D and Table 1D). Alder rhizosphere had a higher relative abundance of *Hypocrea* sp. (median abundance: 0.0041%) and *Thelephora* sp. (median abundance: 0.0088%) compared to birch and spruce rhizosphere, while *Mollisia* were relatively more abundant (median abundance: 0.0084%) in birch rhizosphere (Fig. 9).

When considering the 100 most abundant OTUs in each VDC, and the 100 most abundant OTUs associated with each plant species, some unique OTUs were observed (Tables S5 and S6). Most VDC presented unique OTUs: VDC-1 (e.g., *Leptospirillum* sp., *Acidiphilium* sp.), VDC-2 (e.g., *Terracidiphilus* sp.), VDC-3 (e.g., *Chryseolina* sp., *Sphingomonas* sp.), and VDC-6 (e.g., *Rhizobium* sp., *Rhodoplanes* sp.) (Table S5A). Fungal communities showed more unique OTUs in each VDC, compared to bacterial communities, specifically: VDC-1 (e.g., *Acidomyces* sp., *Sclerotinia* sp.), VDC-2 (e.g., *Peziza* sp., *Gymnopilus* sp.), VDC-3 (e.g., *Lanzia* sp., *Podospora* sp.), VDC-4 (e.g., *Clitopilus* sp., *Hyphodontia* sp.), VDC-5 (e.g., *Kalaharituber* sp., *Phacidium* sp.), and VDC-6 (e.g., *Apodus* sp., *Entoloma* sp.) (Table S6A). In plant species, some unique bacterial OTUs also were noted in *Alnus rugosa* (e.g., *Chryseolinea* sp, *Phaselicystis* sp.), and *Betula papyrifera* (e.g., *Pseudomonas* sp., *Terracidiphilus* sp.) (Table S5A). Fungal communities showed more unique OTUs in each of the plant species, compared to bacterial communities. Unique fungal OTUs included the following in plant species: *Alnus rugosa* (e.g., *Apodus* sp., *Peziza* sp.), *Betula papyrifera* (e.g., *Gymnopilus* sp., *Clitopilus* sp.), and *Picea* sp. (e.g., *Podospora* sp., *Schizosaccharomyces* sp.) (Table S6B).

**Figure 10:**
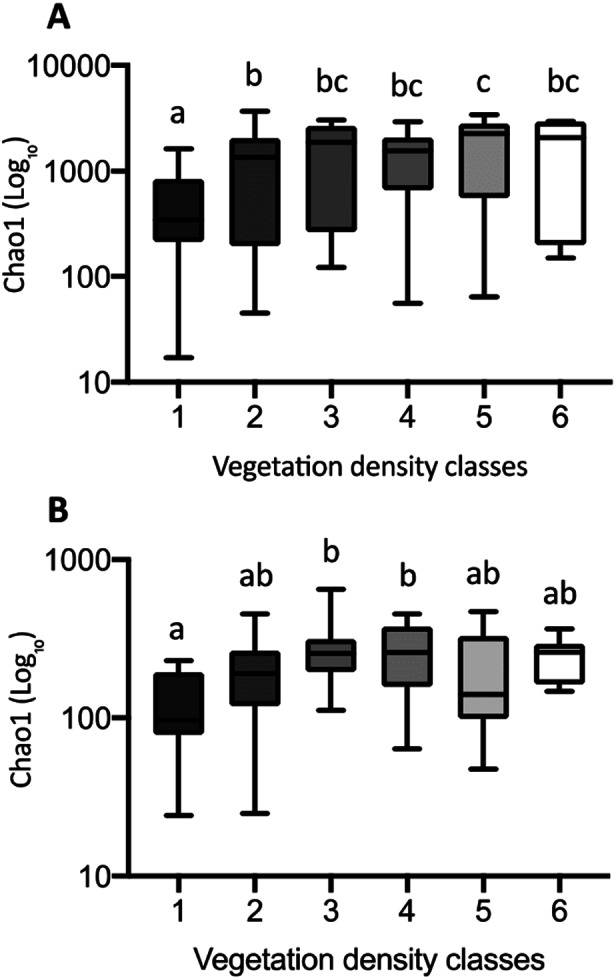
Bacterial (A) and fungal (B) richness calculated using the Chao1 index. The upper and lower edges of the boxes represent respectively the 75th and 25th percentiles. The upper and lower whiskers represent the maximum and minimum values. Different letters represent significant differences (*p*-value < 0.05) between vegetation density classes after Dunn’s (A) and Tukey’s (B) multicomparison tests.

The Chao1 index of richness in microbial population biodiversity showed that bacterial richness in tailings increased significantly in the presence of vegetation, compared to the barren VDC-1 (Fig. 10A). Indeed, bacterial richness in VDC-2 to VDC-6 was 2.5-fold to 3.5 fold higher compared to VDC-1 (VDC-1: 525.7, VDC-2: 1327.1, VDC-3: 1601.9, VDC-4: 1399.5, VDC-5: 1843.8 and VDC-6: 1524.8). We also observed that the level of bacterial diversity in tailings from VDC-2 to VDC-4 was comparable to that of the forest soils of VDC-5 and VDC-6. Fungal richness in diversity did not differ significantly between VDC-1, VDC-2, VDC-5, and VDC-6 but was, however significantly higher in VDC-3 and VDC-4 compared to VDC-1 (Fig. 10B). In each VDC, bacterial diversity was higher compared to that of fungal diversity (3.5-fold to 9.2-fold).

## Discussion

### Vegetation density classes and plant species as drivers of microbial communities

Our results showed that the vegetation density classes (VDC) and plant species (p_s) significantly influenced the structure of the microbial (bacterial and fungal) communities present on the study site (Figs. 2 and 3). The vegetation density classes presented in this paper ranged from a total absence of plants (VDC-1) to a boreal forest, unaffected by human activities (VDC-6). In a mining context, acidic and poor-nutrient tailings make plant establishment difficult, leading to a low carbon concentration in soil, which was observed on tailings (VDC-1, 2, 3 and 4). These VDC had an average organic matter content of 2.1% compared to 22.39% for VDC-5 and 6 (Table S2). The presence of plants is known to change soil properties through the secretion of root exudates composed of organic acids, H ^+^, chelators, and phytosiderophores, which influence pH, the stability of aggregates, and nutrient availability (Anger & Caron, 1998; Gould et al., 2016; Philippot et al., 2009; Van der Putten et al., 2013). The absence of vegetation in VDC-1 leads to a different soil structure and lack of root exudates, which are truly important in the structure of the bacterial communities (Chaparro et al., 2013; Marschner et al., 2001). All these factors could explain why VDC and plant species were the main factors structuring microbial communities in RDAs, as well as the results observed in PCoAs where VDC-1 showed the greatest difference in the bacterial community structure in comparison to all other VDC (Figs. 2 and 3A and Table 1A). Our study also showed that fungal communities were different in the boreal forest soils (VDC-5 and 6) compared to those found on the tailings deposit (VDC-1 to 4) (Fig. 3B and Table 1B). This might be due to the differences in soil carbon levels. A study conducted by Liu et al. (2015a) as well as Sun et al. (2016) reported that carbon concentration in soil was indeed one of the major determinants of fungal community structure.

Several studies have shown that plant species is one of the major determinants of the microbial composition in the rhizosphere soil (Cavaglieri, Orlando & Etcheverry, 2009; Chaparro et al., 2013). Root exudates vary according to plant species, age of the plant, its state of stress, as well as its stage of development (Cavaglieri, Orlando & Etcheverry, 2009; Chaparro et al., 2013; Turner, James & Poole, 2013). It was also showed by Prescott & Grayston (2013), as well as Urbanovà, Šnajdr & Baldrian (2015) that trees mostly influenced composition of fungal and bacterial communities. Our results also showed differences in the rhizospheres of different plant species. PCoA results showed significant differences of bacterial communities in the rhizosphere soil of alder compared to birch and different fungal communities in the rhizosphere soil of alder compared to spruce (Fig. 3). We measured the highest relative abundance of Actinobacteria in alder rhizosphere (4.68%) in comparison to birch (3.30%) and spruce (3.74%) rhizospheres (Fig. 4 and Table S7A). As mentioned previously, plant species can harbour a rhizomicrobiome that is specifically beneficial (Berendsen, Pieterse & Bakker, 2012). The fact that alders establish a root nodule-forming symbiosis with Actinobacteria of the genus *Frankia* might explain the higher relative abundance of Actinobacteria in their rhizosphere. Additionally, this symbiosis requires specific nutrients (i.e., Mo, Mg, Fe and P) to support its nitrogen fixation activity, which might in turn influence the plant-microbe associations that tend to develop (Bélanger, Bellenger & Roy, 2013; Kabata-Pendias, 2001; Ngom et al., 2016).

### Bacteria associated with vegetation density classes and plant species

This study revealed that the six most abundant classes of bacteria for each VDC and p_s were Gammaproteobacteria, Deltaproteobacteria, Alphaproteobacteria, Planctomycetacia, Acidobacteria, and Actinobacteria and represented approximately 50% of the microbial community classes (Fig. 4 and Table S7A). A study conducted by Mendez, Neilson & Maier (2008) reported that the main bacterial classes associated with iron-rich tailings with low pH (2.7) to moderate pH (5.7) were Gammaproteobacteria, Alphaproteobacteria, Acidobacteria, Actinobacteria, *Nitrospira* and Firmicutes, which corroborates some of our results. Hur et al. (2011) showed that zinc mine tailings with low pH, low organic matter as well as high Zn, Cd and Pb concentrations were dominated by Proteobacteria, Actinobacteria and Acidobacteria. The studies done by Hur et al. (2011) as well as Mendez, Neilson & Maier (2008) corroborate what was observed on the studied mine site, that Proteobacteria, Acidobacteria and Actinobacteria were the dominant taxa colonizing mine tailings.

As mentioned above, the rhizosphere soil is the nutrient rich, high biodiversity zone surrounding plant roots. The rhizosphere soil is a beneficial niche for many key microorganisms (Prashar, Kapoor & Sachdeva, 2014). In VDC-1 where there was little to no plant colonization and where pH was the lowest (3.9) compared to other VDC, we found the highest relative abundance of the chemoautotrophs *Acidiferrobacter* sp. and *Leptospirillum* sp. This could be expected since *Acidiferrobacter* sp. derive their energy through the oxidation of various inorganic compounds and *Leptospirillum* sp. does so through the oxidation of iron, which is present in high concentrations (31,000 ppm) in VDC-1 (Table S2) (Tyson et al., 2000). The low organic matter content of the tailings (average of 2.1% in (VDC1 to 4) (Gagnon et al., 2020) likely limited the development of heterotrophic microbial communities in tailings. This could have also contributed to the relative abundance of chemoautotrophs in VDC-1. Such chemoautotrophic organisms tend to be less active in the presence of plants because the latter secrete many carbon-rich molecules, which allows heterotrophs to dominate in their presence (Bais et al., 2006; Turner, James & Poole, 2013).

It is known that PGPR organisms facilitate plant growth in abiotic-stressed environments (Grandlic et al., 2008; Sasse, Martinoia & Northen, 2018; Thijs et al., 2016; Weyens et al., 2009; Zhuang et al., 2007). Berendsen, Pieterse & Bakker (2012), as well as Santoyo et al. (2016), showed that plants could select and shape their rhizomicrobiome as a function of their homeostatic states and for meeting their needs. Plants growing in metal-contaminated soils have different rhizomicrobiomes compared to plants growing in non-contaminated soils; they recruit beneficial microorganisms to support their growth and limit their metal uptake (Sasse, Martinoia & Northen, 2018; Thijs et al., 2016). Microorganisms in the rhizosphere soil can stimulate plant growth by providing nitrogen, solubilizing phosphorus, producing phytohormones (i.e., auxins, cytokinins), synthesizing siderophores, and protecting plants from pathogens (Glick, 2010; Prashar, Kapoor & Sachdeva, 2014).

We observed the presence of nitrogen-fixing, symbiotic organisms such as *Bradyrhizobium* sp., *Frankia* sp., and *Rhizobium* sp. in VDC-2, 3, and 4. These nitrogen fixers may have improved the competitiveness of plants harbored in these nitrogen-poor substrates (Bais et al., 2006; Ma et al., 2011; Mus et al., 2016; Wani, Khan & Zaidi, 2007) as VDC-2 to 4 contained less than 0.25 ppm N (Table S2). *Bradyrhizobium* sp. have been reported to benefit plants growing in metal-contaminated soil. For example, *Bradyrhizodium* sp. (*vigna*) was shown to improve growth, nodulation, nitrogen content, and reduce the uptake of Ni and Zn in green gram (*Vigna radiata)*. This occurred through the production of indole-3-acetic acid, siderophores and ammonia (Ma et al., 2011; Wani, Khan & Zaidi, 2007).

Despite many PGPR traits reported in *Pseudomonas* sp. in contaminated soils (i.e., mine tailings), our study showed (Fig. 6E) that the relative abundance of *Pseudomonas* sp. was higher in boreal forest soils (VDC-5 and 6) compared to that of the tailings storage area (VDC-1 to 4) (Glick, 2010; Ma et al., 2011). *Pseudomonas* sp. are known for their production of organic acids that solubilize mineral phosphorus, and the production of phosphatase/phytase that hydrolyzes phosphate-organic compounds, increasing the availability of this limiting nutrient in soils (Hunter, Teakle & Bending, 2014; Richardson et al., 2009; Vacheron et al., 2013).

In our study, average phosphate concentrations were not systematically higher in boreal forest soils (VDC-5 and VDC-6; 90.8 and 57.9 ppm, respectively) compared to the tailings (VDC-1 to VDC-4; 18.7, 25.8, 62.2, and 35.1 ppm, respectively) however, higher phosphate concentrations did tend to occur in denser vegetation (VDC-3 to VDC-6) (Table S2). Soil phosphate concentrations in VDC-6 might be influenced by the rapid uptake of phosphate by mature trees, as the nutrient becomes available. Our observations are therefore not contradictory; the likely higher demand for phosphorus in the boreal forest vegetation could explain the higher abundance of *Pseudomonas* sp. in these soil (VDC-5 and 6), compared to mine tailings (VDC-1 to 4).

The nitrogen demand of alders is higher than that of other plant species. Despite other tree species, the absorption of nutrient by alder (N and P) is approximately in the same proportion, which could explain the high nitrogen demands by this species to support growth. To meet this high nitrogen demand, plant recruitment of beneficial microorganisms in the rhizosphere soil is primary (Lõhmus et al., 2006). *Bradyrhizobium* sp. were known for their symbiotic association with legume plant, and to promote nodulation in these plants. In addition, like other rhizobia, *Bradyrhizobium* sp. also have the capacity to fix atmospheric nitrogen and to make this nutrient available for other organisms (Saharan & Nehra, 2011). There is little literature on recruitment of *Bradyrhizobium* sp. by alder, but it is possible that this microorganism could be favorably recruited by alder (in comparison to birch and spruce) to improve its nitrogen acquisition. Furthermore, few authors have reported the possible role of *Haliangium* sp. as PGPR organisms. However, Kundim et al. (2003) as well as Ma et al. (2018) do mention that *Haliangium* sp. secrete antifungal molecules that limit the development of phytopathogens. The highest relative abundance of *Bradyrhizobium* sp. and *Haliangium* sp. in alder rhizosphere soil could also be explained by the different bacterial selection between different plant species (Lei et al., 2018; Urbanovà, Šnajdr & Baldrian, 2015).

In essence, our results indicate that bacterial richness was significantly lower in VDC-1 compared to all other VDC, including those of the natural environment (VDC-5 and VDC-6). Considering the increase of vegetation density as an indicator of environmental recovery, these results strongly suggest that bacterial diversity promptly recovers to natural levels following the establishment of vegetation.

### Fungi associated with vegetation density classes and plant species

The ectomycorrhizal (ECM) fungi, arbuscular fungi (AM) and dark septate endophytes (DSE) are reported to enhance the phytoremediation potential of plants through elemental cycling and fungal-metal interactions (Deng & Cao, 2017; Pirttilä & Frank, 2011). ECM fungi include an estimated 6000 species (mostly Basidiomycetes), and establish symbiosis with only 5% of terrestrial plants, in few woody plant families and genera (i.e., *Pinaceae*, *Fagaceae*, *Betula* sp., *Populus* sp., and *Alnus* sp.), AM fungi comprise approximately 150 species of Zygomycetes (Deng & Cao, 2017). They are associated with herbaceous plants, and various woody plant families (Deng & Cao, 2017; Fortin, Plenchette & Piché, 2015; Landeweert et al., 2001; Mandyam & Jumpponen, 2005). The DSE fungi are reported to establish symbiosis with over 600 plant species, including plants that were not reported to be mycorrhizal. They are present in the rhizosphere soil and roots of plants colonizing metal-contaminated sites, and they are characterised as conidial and sterile fungal endophytes, which form melanised inter- and intra-hyphal structures (Mandyam & Jumpponen, 2005; Upson et al., 2009). Many fungal taxa are reported to establish symbiosis with plants colonizing metal-laden sites. For example, strains of genera *Trichoderma* sp., *Fusarium* sp., *Aspergillus* sp., and *Cladosporium* sp., colonized *Portulaca* plant, a heavy metal hyperaccumulator (Deng et al., 2014). *Penicillum* spp. and *Trichoderma* spp. are also the most frequently isolated fungi that attenuate heavy metal stress in plants (Babu et al., 2014a; Babu et al., 2014b; Deng & Cao, 2017; Khan et al., 2014).

ECM fungi can play a role in metal housekeeping through mechanisms such as precipitation, chelation, cell-wall binding, and the binding of metals by organic acids, polyphosphates, peptides and their transport through intracellular compartments (Colpaert et al., 2011). Moreover, glomalin (a glycoprotein) produced by some AM fungi increase heavy metal binding, reducing the uptake of heavy metals by the host plants (Bano & Ashfaq, 2013). In addition, ECM and AM fungi are involved in contaminant detoxification and mediate the nutritional status of heavy metals in plants (Barea, Azcon & Azcon-Aguilar, 2002; Harms, Schlosser & Wick, 2011; Luo et al., 2014; Thijs et al., 2016).

DSE are known to enhance mineral uptake of host plants, increase the utilization of various organic pools and modification of host water uptake. Indeed, the high melanisation of DSE allows them to resist severe drought and heat and increase plant growth under such conditions (Kilvin, Emery & Rudgers, 2013; Mandyam & Jumpponen, 2005; Pirttilä & Frank, 2011; Zhang et al., 2008).

Liu et al. (2015a) reported a shift in fungal communities as a function of organic carbon content in soils: the abundance of Agaricomycetes decreased when more organic carbon was present, and when there was an increase of *Incertae sedis.* Our results corroborate this; the relative abundance of Agaricomycetes in mine tailings (VDC-1 to 4) (average ∼20%) is higher than its relative abundance in boreal forest soil (VDC-5 and 6) (average ∼15%). We also observed, as Liu et al. (2015a), a higher relative abundance of *Incertae sedis* in boreal forest soils (VDC-5 and 6) (∼16%) compared to that in mine tailings (VDC-1 to 4) (∼5%) (Fig. 4 and Table S7B) . The higher organic matter content in boreal forest could also explain our PCoA results that showed a difference in fungal communities in VDC-5 and 6 in comparison to those in mine tailings soils (VDC-1 to 4) (Fig. 3B and Table 1B).

In our study, ECM *Hypocrea* sp. were associated more with plant rhizospheres from VDC-2 to 4, and could potentially be beneficial to plants on such sites. As an example, Morales-Barrera & Cristiani-Urbina (2015) found that *Hypocrea tawa* could be useful to detoxify Cr(VI)-contaminated wastewaters because of its capacity to reduce hexavalent chromium (Cr(VI)) to a much less toxic trivalent chromium form (Cr(III)). *Hypocrea* can solubilize metals (i.e., Cr, Ni, Cu and Pb) and, with other microorganisms, could immobilize these same heavy metals following their solubilisation (Congeevaram et al., 2007; Kumari et al., 2015). Based on the previous studies, *Hypocrea* sp. could have alleviated heavy metal stress, helping host plants grow on the tailings storage site.

Our study revealed a higher relative abundance of *Mollisia* sp. in VDC-1 and 2. This could be explained by the fact that *Mollisia* sp. is known for its high metal tolerance (Pirttilä & Frank, 2011), and that the low organic matter content in these VDC likely provided conditions allowing high bioavailability of metals (Table S2).

Our study also found a greater relative abundance of Zygomycota fungi (*Dissophora* sp. and *Mortierella* sp.) in boreal forest soil (VDC-5 and 6) compared to tailings (VDC-1 to 4). These observations corroborate those of Liu et al. (2015a) and Sun et al. (2016) according to which the relative abundance of Zygomycota (and specifically *Mortierella* sp.) increased with soil carbon content. The organic matter content of VDC-5 and 6 were on average 22.4%, while they were 2.1% on the tailings storage area VDC (Fig. 7 and Table S2).

The capability of plants species to shape their rhizosphere soil microbiome to adapt to various environmental conditions can lead to significant differences in the relative abundance of diverse fungi and bacteria (Berendsen, Pieterse & Bakker, 2012; Sasse, Martinoia & Northen, 2018). Prescott & Grayston (2013) and Urbanovà, Šnajdr & Baldrian (2015) reported that fungal communities in soil were largely explained by dominant trees. Urbanovà, Šnajdr & Baldrian (2015) reported that different proportions of arbuscular and ectomycorrhizal fungi were found under different tree species. In our study, we showed that birch rhizosphere present a higher relative abundance of the DSE fungus *Mollisia* sp. compared to the rhizosphere soil of alder and spruce (Fig. 9A). This finding is similar to that reported by Fernández-Miranda Cagigal (2017), where DSE were the dominant fungi of healthy fine root of, amongst other species, *Betula pubescens*.

Globally, we observed that fungal richness was not as dependent upon the presence or density of vegetation as was bacterial diversity. Fungal diversity did not differ significantly between VDC-1 to VDC-4, compared to that of boreal forest soils (VDC-5 and VDC-6). While bacterial richness in diversity increased following vegetation establishment, that of fungal communities remained relatively stable. These observations corroborate those of Sun et al. (2017) which found that bacterial diversity was 7.0- to 7.5-fold greater compared to fungal diversity in a recovering forest ecosystem.

## Conclusions

Our results indicate that vegetation density classes (VDC) and plant species best explained the bacterial and fungal community structures. Indeed, PCoA showed significant differences in total microbial communities between the various VDC as well as plant species. These two parameters are intimately related, since the plants, by their root exudates, are able to select the type of microorganisms composing their rhizospheres. Moreover, the plants that colonize these soils change them differently, from one VDC to another, thus producing different physical and chemical properties. These may or may not stimulate plants to structure their rhizosphere soil microbial communities in different ways.

The capability of complex plant assemblages to colonize a tailings storage area with these properties is a demonstration of autonomous recovery following human disturbance. We observed that as vegetation density increased and began in some sectors to resemble that of the adjacent natural forest, microbial communities associated with alder, spruce, and birch varied. Indeed, while plant species is a known determinant of microbial community composition in the rhizosphere, this study revealed that the parameter of vegetation density also explained community structure. This is an important observation since the capability of nutrient-poor substrates to sustain plant growth can be improved by the presence of bacterial and fungal communities capable of weathering minerals and increase water and nutrient acquisition in plants. Microbial communities and more microbial diversity can improve survival and development of plants. They can contribute directly and indirectly to resilience of these recovering environments. By extension, it is worthwhile to consider planting biodiverse plant thickets in reclamation efforts. While our study begins to shed light into the associations, and specific fungal and bacterial taxa, that were found to thrive in this particular environment, subsequent studies will be required to deepen our understanding of the specific plant-microbe associations we observed. Ultimately, this will help us better harness plant-microbe and plant-plant associations to improve and accelerate the ecological restoration of anthropized environments.

##  Supplemental Information

10.7717/peerj.10109/supp-1 Supplemental Information 1 Number of replicates (*n* =) associated with each figure

10.7717/peerj.10109/supp-2 Supplemental Information 2 Scale of concentration of various environmental properties measured in soils Water and organic matter content were reported in percentage (%) and the metal results in ppm (µg_metal.g_dryweight^−1^). Avg: average, Min: minimum, Max: maximum, MD: mean deviation, Med: median, VDC: vegetation density classes.

10.7717/peerj.10109/supp-3 Supplemental Information 3 Primers used for the amplification of bacterial (16S rRNA) and fungal (ITS) sequences

10.7717/peerj.10109/supp-4 Supplemental Information 4 Significant axes revealed in the redundancy analysis of soil variables effect on bacterial and fungal community composition vdc: vegetation density classes, p_s: plant species.

10.7717/peerj.10109/supp-5 Supplemental Information 5 Venn’s table showing shared and unique bacterial OTUs Venn’s table showing shared and unique bacterial OTUs, from the 100 most abundant taxa, between vegetation density classes (A) and plant species (B). The letter in front of the taxon represents the maximum depth of taxonomy (g: genus, f: family, c: class, o: order, p: phylum, k: kingdom).

10.7717/peerj.10109/supp-6 Supplemental Information 6 Venn’s table showing shared and unique fungal OTUs Venn’s table showing shared and unique fungal OTUs, from the 100 most abundant taxa, between vegetation density classes (A) and plant species (B). The letter in front of the taxon represents the maximum depth of taxonomy (g: genus, f: family, c: class, o: order, p: phylum, k: kingdom).

10.7717/peerj.10109/supp-7 Supplemental Information 7 Relative abundance of transcripts (%) of the 6 most abundant bacteria (A) and fungi (B) classes between the different vegetation density classes and plant species SD: standard-deviation, VDC: vegetation density classes, p_s: plant species.

## Additional Information and Declarations

### Field Study Permissions

The following information was supplied relating to field study approvals (i.e., approving body and any reference numbers): The owners of the mine site granted access to our research team through an agreement between the Université de Sherbrooke and the mine owners which allows the scientific study of the area surrounding the mine.

### DNA Deposition

The following information was supplied regarding the deposition of DNA sequences: Sequencing data are available at the NCBI Sequence Read Archive (SRA): PRJNA634113.

### Data Availability

The following information was supplied regarding data availability: The data are available at Figshare: Gagnon, Vanessa; Wasserscheid, Jessica; Rodrigue-Morin, Michaël; Tremblay, Julien; Champagne, Julie; Bellenger, Jean-Philippe; et al. (2020): code_dataset. figshare. Dataset. https://doi.org/10.6084/m9.figshare.12530534.v1.
