# Supplementary material for: Vegetation drives the structure of active microbial communities on an acidogenic mine tailings deposit
Source: PeerJ. 2020 Oct 21;8:e10109. doi: 10.7717/peerj.10109 (PMC7585372; doi:10.7717/peerj.10109)
Supplement: Supplemental Information 1 [file peerj-08-10109-s001.docx]

**Table S1.** Number of replicates (n=) associated with each figure.

**Figure 2A and 2C**, n= 173, **Figure 2B and 2D**, n= 118.

**Figure 3.**

|  | **VDC-1** | **VDC-2** | **VDC-3** | **VDC-4** | **VDC-5** | **VDC-6** |
| --- | --- | --- | --- | --- | --- | --- |
| **A** (n=) | 37 | 34 | 38 | 32 | 25 | 7 |
| **B** (n=) | 15 | 35 | 16 | 31 | 15 | 6 |

**Figure 3.**

|  | **Alder** | **Birch** | **Spruce** |
| --- | --- | --- | --- |
| **C** (n=) | 31 | 37 | 36 |
| **D** (n=) | 25 | 34 | 23 |

**Figure 4A.**

|  | **VDC-1** | **VDC-2** | **VDC-3** | **VDC-4** | **VDC-5** | **VDC-6** |
| --- | --- | --- | --- | --- | --- | --- |
| (n=) | 38 | 38 | 41 | 36 | 30 | 9 |

**Figure 4B**.

|  | **Alder** | **Birch** | **Spruce** |
| --- | --- | --- | --- |
| (n=) | 36 | 41 | 38 |

**Figure 5A.**

|  | **VDC-1** | **VDC-2** | **VDC-3** | **VDC-4** | **VDC-5** | **VDC-6** |
| --- | --- | --- | --- | --- | --- | --- |
| **A** (n=) | 15 | 35 | 16 | 31 | 23 | 6 |

**Figure 5B.**

|  | **Alder** | **Birch** | **Spruce** |
| --- | --- | --- | --- |
| (n=) | 25 | 33 | 24 |

**Figure 6.**

|  | **VDC-1** | **VDC-2** | **VDC-3** | **VDC-4** | **VDC-5** | **VDC-6** |
| --- | --- | --- | --- | --- | --- | --- |
| (n=) | 37 | 34 | 38 | 32 | 25 | 7 |

**Figure 7.**

|  | **VDC-1** | **VDC-2** | **VDC-3** | **VDC-4** | **VDC-5** | **VDC-6** |
| --- | --- | --- | --- | --- | --- | --- |
| (n=) | 15 | 35 | 16 | 31 | 15 | 6 |

**Figure 8.**

|  | **Alder** | **Birch** | **Spruce** |
| --- | --- | --- | --- |
| (n=) | 31 | 37 | 36 |

**Figure 9.**

|  | **Alder** | **Birch** | **Spruce** |
| --- | --- | --- | --- |
| (n=) | 25 | 34 | 23 |

**Figure 10.**

|  | **VDC-1** | **VDC-2** | **VDC-3** | **VDC-4** | **VDC-5** | **VDC-6** |
| --- | --- | --- | --- | --- | --- | --- |
| **A** (n=) | 37 | 34 | 38 | 32 | 25 | 7 |
| **B** (n=) | 14 | 35 | 16 | 30 | 15 | 6 |
