# Supplementary material for: Vegetation drives the structure of active microbial communities on an acidogenic mine tailings deposit
Source: PeerJ. 2020 Oct 21;8:e10109. doi: 10.7717/peerj.10109 (PMC7585372; doi:10.7717/peerj.10109)
Supplement: Supplemental Information 2 — Water and organic matter content were reported in percentage (%) and the metal results in ppm (µg_metal.g_dryweight−1). Avg: average, Min: minimum, Max: maximum, MD: mean deviation, Med: median, VDC: vegetation density classes. [file peerj-08-10109-s002.docx]

**Table S2**. Scale of concentration of various environmental properties measured in soils. Water and organic matter content were reported in percentage (%) and the metal results in ppm (μg_metal_.g_dryweight_^-1^). Avg: average, Min: minimum, Max: maximum, MD: mean deviation, Med: median, VDC: vegetation density classes.

| Environmental properties | VDC-1 | | | VDC-2 | | | VDC-3 | | |
| --- | --- | --- | --- | --- | --- | --- | --- | --- | --- |
|  | Min-Max | Avg ± MD | Med. | Min-Max | Avg ± MD | Med. | Min-Max | Avg ± MD | Med. |
| pH | 2.94-5.65 | 3.88± 0.58 | 3.91 | 3.66-7.34 | 4.95 ± 0.81 | 4.68 | 3.96-8.90 | 7.07±1.66 | 8.11 |
| Water content | 6.27-28.08 | 15.93 ± 5.56 | 15.42 | 2.09-56.65 | 19.21 ± 10.87 | 12.91 | 7.69-32.51 | 18.07±3.99 | 17.22 |
| Organic matter content | 1.00-3.30 | 1.96 ± 0.80 | 1.50 | 1.30-2.30 | 1.87 ± 0.24 | 1.80 | 0.80-3.10 | 1.92 ± 0.59 | 1.70 |
| Total nitrogen | <0.01 | <0.01 | <0.01 | <0.01 | <0.01 | <0.01 | <0.01-0.3 | 0.25 ± 0.05 | 0.25 |
| ^23^Na | 0.30-145.33 | 25.70 ± 25.53 | 10.87 | 0.33-119.20 | 27.54 ± 15.87 | 23.09 | 0.08-139.25 | 41.24 ± 33.30 | 31.01 |
| ^24^Mg | 66.24-4304.56 | 1230.22 ± 593.89 | 1064.63 | 447.35-3965.78 | 1333.02 ± 561.17 | 1037.93 | 389.79-5195.45 | 1959.52 ± 1291.33 | 1448.77 |
| ^27^Al | 116.93-6123.02 | 2061.57 ± 896.49 | 1824.79 | 965.14-7011.41 | 2225.36 ± 651.09 | 1984.27 | 961.87-4919.81 | 2000.86 ± 580.81 | 11879.37 |
| ^31^P | 1.89-156.54 | 18.74 ± 13.39 | 11.27 | 8.25-77.70 | 25.77 ± 13.87 | 18.75 | 7.75-102.96 | 62.15 ± 27.32 | 77.31 |
| ^39^K | 2.76-1260.31 | 248.89 ± 217.29 | 117.95 | 49.92-1377.57 | 292.31 ± 178.15 | 192.04 | 4.45-1319.92 | 453.01 ± 312.74 | 397.49 |
| ^44^Ca | 6.54-4902.04 | 301.26 ± 584.73 | 36.30 | 24.00-14681.10 | 933.20 ± 1146.57 | 168.23 | 9.90-55818.18 | 20979.08 ± 21123.48 | 2340.18 |
| ^47^Ti | 1.09-35.91 | 10.66± 6.22 | 8.86 | 2.14-73.84 | 18.86 ± 14.23 | 13.71 | 1.41-87.75 | 15.55 ±11.58 | 11.05 |
| ^51^V | 0.45-21.61 | 4.00 ± 2.30 | 3.08 | 2.52-17.68 | 4.51 ± 1.58 | 3.69 | 1.23-21.80 | 8.65 ± 4.15 | 8.84 |
| ^52^Cr | 0.33-14.66 | 4.29 ± 2.72 | 2.81 | 1.98-19.00 | 5.18 ± 2.20 | 4.24 | 1.18-20.86 | 5.70 ± 2.74 | 5.08 |
| ^55^Mn | 2.53-162.42 | 57.65 ± 32.63 | 51.80 | 12.27-742.91 | 139.08 ± 121.46 | 68.31 | 10.82-1061.65 | 479.49 ± 426.51 | 338.17 |
| ^57^Fe | 2249.64- 70039.68 | 31018.19 ± 11106.73 | 27298.85 | 19669.09-65200.00 | 34910.89 ± 8183.13 | 32249.10 | 17029.05-66792.45 | 29646.14 ± 10257.42 | 23788.84 |
| ^59^Co | 0.11-13.89 | 2.92 ± 1.67 | 2.56 | 0.78-16.93 | 4.61 ± 3.21 | 2.90 | 0.46-10.40 | 4.36 ± 2.79 | 5.48 |
| ^60^Ni | 0.20-24.42 | 6.57 ± 4.04 | 5.05 | 1.52-15.92 | 6.71 ± 2.80 | 5.58 | 1.02-13.55 | 6.61 ± 3.39 | 6.61 |
| ^65^Cu | 12.26-456.63 | 220.71 ± 83.71 | 201.73 | 113.84-529.86 | 266.28 ± 74.30 | 251.40 | 24.39-6 11.62 | 164.12 ± 117.94 | 139.51 |
| ^66^Zn | 1.37-115.78 | 41.33 ± 17.15 | 34.95 | 19.47-93.40 | 48.51-17.11 | 42.42 | 12.74- 60.17 | 40.44 ± 10.89 | 42.55 |
| ^75^As | 40.16-1610.95 | 386.42 ± 238.56 | 265.25 | 219.10-1950.95 | 487.95 ± 172.03 | 428.86 | 177.29- 1687.26 | 666.56 ± 241.87 | 568.16 |
| ^82^Se | 0.68-7.41 | 4.50 ± 1.18 | 4.74 | 2.65-12.30 | 5.10 ± 1.34 | 4.56 | 0.73-4.63 | 2.35 ± 1.14 | 2.07 |
| ^95^Mo | 1.26-24.49 | 10.76 ± 4.20 | 10.75 | 4.80-32.93 | 12.82 ± 4.55 | 11.19 | 2.45-17.29 | 9.28 ± 2.83 | 8.73 |
| ^107^Ag | 0.32-6.32 | 3.10 ± 1.01 | 2.92 | 1.50-7.84 | 3.32 ± 1.16 | 2.81 | 1.23-3.42 | 2.06 ± 0.46 | 1.93 |
| ^111^Cd | <0.01-0.25 | 0.07 ± 0.04 | 0.070 | 0.01-0.36 | 0.13 ± 0.08 | 0.09 | <0.01-0.47 | 0.15 ± 0.11 | 0.12 |
| ^121^Sb | 0.01-1.69 | 0.55 ± 0.33 | 0.40 | 0.11-1.58 | 0.56 ± 0.25 | 0.50 | 0.09-1.73 | 1.03 ± 0.39 | 1.19 |
| ^137^Ba | 0.82-46.41 | 13.88 ± 9.39 | 9.91 | 1.96-43.91 | 13.50 ± 6.44 | 11.65 | 2.78-52.80 | 23.48 ± 14.97 | 23.42 |
| ^182^W | 0.02-0.51 | 0.17 ± 0.08 | 0.14 | 0.06-0.58 | 0.16 ± 0.05 | 0.14 | 0.03-0.53 | 0.25 ± 0.11 | 0.28 |
| ^205^Tl | 0.09-1.52 | 0.71 ± 0.34 | 0.64 | 0.26-1.51 | 0.61 ± 0.23 | 0.55 | 0.11-0.54 | 0.28 ± 0.08 | 0.25 |
| ^206^Pb | 2.63-54.06 | 20.78 ± 10.29 | 15.67 | 7.20-37.60 | 19.39 ± 6.35 | 18.46 | 5.31-40.62 | 17.52 ± 5.46 | 16.36 |

**Table S3.** Continued.

| Environmental properties | VDC-4 | | | VDC-5 | | | VDC-6 | | |
| --- | --- | --- | --- | --- | --- | --- | --- | --- | --- |
|  | Min-Max | Avg ± MD | Med. | Min-Max | Avg ± MD | Med. | Min-Max | Avg ± MD | Med. |
| pH | 3.61-7.29 | 5.15 ± 0.93 | 5.11 | 5.22-8.85 | 6.96 ± 0.65 | 6.74 | 4.90-6.81 | 6.01 ± 0.53 | 6.08 |
| Water content | 9.82-35.99 | 24.34 ± 5.48 | 25.23 | 9.64-69.04 | 37.30 ± 17.85 | 37.40 | 17.96- 28.69 | 19.59 ± 2.44 | 17.96 |
| Organic matter content | 1.70-2.90 | 2.43 ± 0.33 | 2.50 | 1.30-24.00 | 5.26 ± 3.99 | 2.35 | 15.00-87.30 | 39.52 ± 28.16 | 21.55 |
| Total nitrogen | <0.01 | <0.01 | <0.01 | <0.1-6 | 1.57 ± 1.48 | 0.75 | 20.60-72.00 | 36.58 ± 12.94 | 30.45 |
| ^23^Na | 0.82-94.05 | 31.78 ± 19.33 | 28.08 | 5.80- 143.27 | 39.75 ± 17.85 | 31.12 | 30.72- 91.58 | 38.63 ± 13.24 | 31.12 |
| ^24^Mg | 44.62-5247.93 | 1675.32 ± 836.48 | 1419.78 | 699.26-  6546.56 | 2183.28 ± 922.72 | 1851.77 | 662.66- 5308.62 | 2234.13 ± 1469.63 | 1717.91 |
| ^27^Al | 44.62-6505.54 | 2718.87 ± 1172.09 | 2409.43 | 748.00-  6959.51 | 2673.63 ± 767.70 | 2527.02 | 427.85- 2359.92 | 1908.72 ± 564.00 | 2359.92 |
| ^31^P | 3.59-105.99 | 35.09 ± 16.25 | 31.43 | 22.23- 134.31 | 90.77 ± 24.55 | 93.14 | 12.91- 88.67 | 57.86 ± 19.20 | 60.88 |
| ^39^K | 15.04-  1385.34 | 348.01 ± 198.43 | 298.60 | 315.92-  1720.47 | 991.91 ± 317.75 | 1051.64 | 257.85- 1697.97 | 749.09 ± 477.79 | 419.68 |
| ^44^Ca | 18.90-  35348.15 | 1652.27 ± 2322.12 | 132.69 | 860.93- 49000.00 | 16557.93 ± 9510.98 | 11852.63 | 58.03- 20552.30 | 7862.06 ± 6287.11 | 5818.89 |
| ^47^Ti | 2.56-  167.27 | 19.78 ± 16.73 | 10.75 | 11.32- 41.58 | 19.18 ± 5.06 | 18.24 | 16.91- 72.10 | 31.22 ± 19.80 | 18.24 |
| ^51^V | 1.37-17.66 | 6.34 ± 2.87 | 5.76 | 2.76-18.68 | 11.04 ± 3.60 | 11.52 | 0.92- 17.64 | 7.44 ± 5.02 | 5.88 |
| ^52^Cr | 1.49-20.27 | 8.07 ± 4.55 | 6.60 | 1.52-26.99 | 9.48 ± 3.58 | 9.52 | 0.96- 26.50 | 7.36 ± 4.78 | 6.06 |
| ^55^Mn | 4.19-  1013.64 | 169.80 ± 163.19 | 78.81 | 146.99- 1120.49 | 626.31 ± 240.98 | 593.20 | 35.51- 1421.68 | 326.78 ± 273.72 | 233.87 |
| ^57^Fe | 445.75-  84649.12 | 43251.75 ± 15256.36 | 44452.42 | 2215.20- 24008.10 | 13628.88 ± 4747.87 | 12810.41 | 584.12- 36678.26 | 19984.08 ± 12484.15 | 21215.43 |
| ^59^Co | 1.00-14.94 | 5.11 ± 2.84 | 4.57 | 1.55-17.16 | 7.31 ± 2.53 | 7.33 | 0.70-4.94 | 3.40 ± 1.63 | 4.57 |
| ^60^Ni | 2.08-32.25 | 13.16 ± 8.10 | 9.23 | 2.94-26.40 | 13.36 ± 5.89 | 12.95 | 2.17-31.44 | 14.64 ± 9.02 | 13.15 |
| ^65^Cu | 40.31- 407.06 | 229.95 ± 72.31 | 235.64 | 12.51- 56.77 | 33.06 ± 9.83 | 31.32 | 20.46- 311.24 | 69.64 ± 60.40 | 33.00 |
| ^66^Zn | 4.20- 125.46 | 58.70 ± 22.92 | 57.07 | 15.77- 126.93 | 60.90 ± 22.06 | 53.07 | 37.47- 130.07 | 96.08 ± 21.88 | 98.53 |
| ^75^As | 43.32-  873.94 | 379.91 ± 114.13 | 350.32 | 4.11- 1376.10 | 473.80 ± 304.81 | 443.40 | 3.10- 276.87 | 62.51 ± 53.59 | 37.51 |
| ^82^Se | 0.69-9.59 | 4.94 ± 1.77 | 4.62 | <0.01-2.78 | 1.43 ± 0.50 | 1.50 | 0.55-3.66 | 1.83 ± 0.95 | 1.43 |
| ^95^Mo | 1.93-29.12 | 11.78 ± 4.18 | 10.77 | 0.36-26.10 | 13.20 ± 6.11 | 12.57 | 0.83-24.82 | 6.05 ± 4.89 | 4.15 |
| ^107^Ag | 0.22-7.73 | 2.95 ± 1.08 | 2.86 | 0.03-3.96 | 1.61 ± 0.92 | 1.45 | 0.08-4.62 | 0.74 ± 0.97 | 0.21 |
| ^111^Cd | 0.02-0.31 | 0.11 ± 0.06 | 0.09 | 0.09-2.48 | 0.28 ± 0.22 | 0.12 | 0.07-0.12 | 0.11 ± 0.01 | 0.12 |
| ^121^Sb | 0.04-4.27 | 0.60 ± 0.33 | 0.47 | 0.01-2.40 | 1.50 ± 0.49 | 1.65 | 0.05-0.87 | 0.26 ± 0.17 | 0.23 |
| ^137^Ba | 4.03-70.05 | 23.01 ± 14.64 | 16.36 | 9.61-75.10 | 37.72 ± 14.42 | 34.15 | 5.57-54.07 | 19.64 ± 8.61 | 16.36 |
| ^182^W | 0.02-0.43 | 0.17 ± 0.08 | 0.14 | 0.03-0.53 | 0.22 ± 0.10 | 0.24 | 0.05-0.20 | 0.10 ± 0.03 | 0.09 |
| ^205^Tl | 0.08-1.95 | 0.68 ± 0.33 | 0.60 | 0.03-0.70 | 0.31 ± 012 | 0.28 | 0.02-0.29 | 0.18 ± 0.09 | 0.20 |
| ^206^Pb | 3.23-37.87 | 17.56 ± 6.07 | 16.70 | 8.18-84.79 | 30.23 ± 13.96 | 20.38 | 13.31- 47.50 | 26.92 ± 8.54 | 26.36 |
