# Supplementary material for: Vegetation drives the structure of active microbial communities on an acidogenic mine tailings deposit
Source: PeerJ. 2020 Oct 21;8:e10109. doi: 10.7717/peerj.10109 (PMC7585372; doi:10.7717/peerj.10109)
Supplement: Supplemental Information 3 [file peerj-08-10109-s003.docx]

**Table S3.** Primers used for the amplification of bacterial (16S rRNA) and fungal (ITS) sequences.

| Primer set | Target | Name | Overhang sequence |
| --- | --- | --- | --- |
| 1 | Bacterial, 16S rRNA (Caporaso et al., 2011) | 515 ill (forward) | 5’ - TCGTCGGCAGCGTCAGATGTGTATAAGAGACAG- 3’ |
|  |  | 806 ill (reverse) | 5’ - GTCTCGTGGGCTCGGAGATGTGTATAAGAGACAG - 3’ |
| 2 |  | 515 ill/1 (forward) | 5’ - TTCGTCGGCAGCGTCAGATGTGTATAAGAGACAG - 3’ |
|  |  | 806 ill/1 (reverse) | 5’ - TGTCTCGTGGGCTCGGAGATGTGTATAAGAGACAG - 3’ |
| 3 |  | 515 ill/2 (forward) | 5’ - ACTCGTCGGCAGCGTCAGATGTGTATAAGAGACAG - 3’ |
|  |  | 806 ill/2 (reverse) | 5’ - ACGTCTCGTGGGCTCGGAGATGTGTATAAGAGACAG - 3’ |
| 4 |  | 515 ill/3 (forward) | 5’ - CTATCGTCGGCAGCGTCAGATGTGTATAAGAGACAG - 3’ |
|  |  | 806 ill/3 (reverse) | 5’ - CTAGTCTCGTGGGCTCGGAGATGTGTATAAGAGACAG - 3’ |
| 5 | Fungal, ITS (Kendall & Rygiewic, 2005) | ITS F illu forward) | 5’- TCGTCGGCAGCGTCAGATGTGTATAAGAGACAG - 3’ |
|  |  | 58A2R-illu (reverse) | 5’ - GTCTCGTGGGCTCGGAGATGTGTATAAGAGACAG - 3’ |
| 6 |  | ITS F illu/1 (forward) | 5’ - TTCGTCGGCAGCGTCAGATGTGTATAAGAGACAG - 3’ |
|  |  | 58A2R-illu/1 (reverse) | 5’ - TGTCTCGTGGGCTCGGAGATGTGTATAAGAGACAG - 3’ |
| 7 |  | ITS F illu/2 (forward) | 5’ - AGTCGTCGGCAGCGTCAGATGTGTATAAGAGACAG - 3’ |
|  |  | 58A2R-illu/2 (reverse) | 5’ - AGGTCTCGTGGGCTCGGAGATGTGTATAAGAGACAG - 3’ |
| 8 |  | ITS F illu/3 (forward) | 5’ - GTATCGTCGGCAGCGTCAGATGTGTATAAGAGACAG - 3’ |
|  |  | 58A2R-illu/3 (reverse) | 5’ – GTAGTCTCGTGGGCTCGGAGATGTGTATAAGAGACAG - 3’ |

**SUPPLEMENTAL TABLE S3 REFERENCES**

Caporaso JG, Lauber CL, Walters WA, Berg-Lyons D, Lozupone CA, Turnbaugh PJ, Fierer N, Knight R, 2011. Global patterns of 16S rRNA diversity at a depth of millions of sequences per sample. *Proceedings of the National Academy of Sciences of the United States of America* **108**(1):4516-4522 DOI: 10.1073/pnas.1000080107

Kendall JM, Rygiewick PT. 2005. Fungal-specific primers developed for analysis of the ITS region of environmental DNA extracts. *BMC Microbiology* **5**(28): 1-11 DOI: [10.1186/1471-2180-5-28](https://doi.org/10.1186/1471-2180-5-28)
