# Supplementary material for: Vegetation drives the structure of active microbial communities on an acidogenic mine tailings deposit
Source: PeerJ. 2020 Oct 21;8:e10109. doi: 10.7717/peerj.10109 (PMC7585372; doi:10.7717/peerj.10109)
Supplement: Supplemental Information 4 — vdc: vegetation density classes, p_s: plant species. [file peerj-08-10109-s004.docx]

**Table S4.** Significant axes revealed in the redundancy analysis of soil variables effect on bacterial and fungal community composition. vdc: vegetation density classes, p_s: plant species.

**Figure 2A.**

| Significant axes | *p*-value |
| --- | --- |
| vdc | 0.001 |
| pH | 0.001 |
| Ca | 0.001 |
| p_s | 0.008 |
| Ag | 0.023 |

**Figure 2B.**

| Significant axes | *p*-value |
| --- | --- |
| vdc | 0.001 |
| pH | 0.001 |
| p_s | 0.010 |
| Na | 0.010 |
| Ti | 0.001 |
| W | 0.008 |
| Fe | 0.007 |

**Figure 2C.**

| Significant axes | *p*-value |
| --- | --- |
| vdc | 0.001 |
| Ca | 0.001 |
| p_s | 0.007 |
| Mn | 0.002 |
| Cu | 0.010 |
| Fe | 0.031 |

**Figure 2D.**

| Significant axes | *p*-value |
| --- | --- |
| vdc | 0.001 |
| pH | 0.001 |
| p_s | 0.001 |
| Cu | 0.006 |
| Ti | 0.006 |
| W | 0.024 |
| Cd | 0.031 |
| Na | 0.031 |
| Cr | 0.043 |
