# Supplementary material for: Vegetation drives the structure of active microbial communities on an acidogenic mine tailings deposit
Source: PeerJ. 2020 Oct 21;8:e10109. doi: 10.7717/peerj.10109 (PMC7585372; doi:10.7717/peerj.10109)
Supplement: Supplemental Information 5 — Venn’s table showing shared and unique bacterial OTUs, from the 100 most abundant taxa, between vegetation density classes (A) and plant species (B). The letter in front of the taxon represents the maximum depth of taxonomy (g: genus, f: family, c: class, o: order, p: phylum, k: kingdom). [file peerj-08-10109-s005.docx]

**Table S5.** Venn’s table showing shared and unique bacterial OTUs, from the 100 most abundant taxa, between vegetation density classes (A) and plant species (B). The letter in front of the taxon represents the maximum depth of taxonomy (g: genus, f: family, c: class, o: order, p: phylum, k: kingdom).

**A**

**Vegetation density**

**classes**

**Number**

**of OTUs**

**OTUs**

_1 _2 _3 _4 _5 _6

77

f

__

MSB

-

4B10; o__

Ktedonobacterales

; p__

Latescibacteria

; g__

uncultured

-

S

imkaniaceae; g__*Haliangium*;

c__

Subgroup

6; o__X35; f__SM2D12; p__Proteobacteria; g__

*Edaphobacter*; g*_*_

*Acidicapsa*; f*_*

_cvE6;

f__

Xanthomonadales

Incertae

sedis; p__

TM6

(Dependentiae

); g__

*Gemmatimonas*

; g__

*Acidibacter*

;

f__DA111; o__Acidimicrobiales; c__

OPB35

soil

group; g__

*Rhizomicrobium*

; f__

Acidobacteriaceae (Subgroup

1

); g__

*Legionella*

; g__

*Aciditerrimonas*

; f__Tepidisphaeraceae; g__SM1A02; g__

*Bdellovibrio*

; c__

Candidatus

Azambacteria; g__

*Gaiella*

; g__

*Coxiella*; g*_*

_

*Acidobacterium*

; f__Fimbriimonadaceae; g__

*Opitutus*

; c__TK10;

g__

*Gemmata*

; o__Obscuribacterales; g__

uncultured

-

Acidobacteriaceae (Subgroup 1

); p__Parcubacteria;

g__

*Chthoniobacter*

; g__

*Aquicella*; f*_*

_ODP1230B8.23; g__

*Pirellula*

; g__H16; g__

Pir4

lineage; o__

SAR324

clade(Marine group B

); g__uncultured

-

Planctomycetaceae; c__Alphaproteobacteria; o__Armatimonadales;

g__

*Singulisphaera*

; g__

*Planctomyces*

; g__

*Candidatus*

*protochlamydia*

; g__

*Candidatus*

*solibacter*;

f*_*

_

Acidobacteriaceae

(Subgroup 1

); g__BD7

-

11CL; f__Planctomycetaceae; p__Chloroflexi;

f__Oligoflexaceae; c__Gammaproteobacteria; g__

*Telmatobacter*

; p__Saccharibacteria; f__P3OB

-

42;

c__OM190; c__KD4

-

96; g__

*Neochlamydia*

; g__

*Acidothermus*

; g__

*Bryobacter*; g*_*

_

*Sorangium*

; f__BIrii41;

p__Omnitrophica; k__Bacteria; f__Planctomycetaceae; f__

env.OPS

17; c__JG37

-

AG

-

4; c__

Subgroup

2;

g__

*Granulicella*

; o__HTA4; o__Ktedonobacterales; f__

0319

-

6G20

_1 _2 _3 _4 _5

4

c

__Pla4

lineage; c__vadinHA49; o__C0119; p__

Woesearchaeota

(DHVEG

-

6

)

_1 _2 _3 _4 _6

1

g__

*Jatrophihabitans*

_1 _2 _4 _5 _6

2

g__

*Pseudomonas*

; g__

*Flavobacterium*

_2 _3 _4 _5 _6

6

g

__

uncultured

-

Cy

tophagaceae; g__

*Nocardioides*

; g__uncultured

-

Chitinophagaceae; f__Bradyrhizobiaceae;

g__

*Mucilaginibacter*

; g__

*Phenylobacterium*

_1 _2 _3 _4

2

g__

*Bryocella*

; g__

*Anaeromyxobacter*

_2 _3 _4 _6

1

g__

*Burkholderia*

*-*

*Paraburkholderia*

_3 _4 _5 _6

1

f

__Elev

-

16S

-

1332

_1 _2 _3

1

f

__mle1

-

27

_2 _5 _6

1

g__

*Phaselicystis*

_4 _5 _6

2

g__

*Ferruginibacter*

; g*_*

_

*Candidatus*

*xiphinematobacter*

_1 _2

2

g__

*Isosphaera*

; p__

FCPU426

_2 _4

1

f__Chitinophagaceae

_3 _5

1

g__

*Geobacter*

_4 _6

1

g__

*Parafilimonas*

_1

11

g__

*Leptospirillum*

;

c__uncultured

; o__

CPla

-

3 termite

group; g__

*Metallibacterium*

; o*_*

_

Acidimicrobiales

;

g__

uncultured

-

Xanth

omonadaceae; g__

*Ferrithrix*

; c__ML635J

-

21; p__

Candidatus

Berkelbacteria;

g__

*Acidiphilium*

; g__

*Acidiferrobacter*

_2

2

g__

*Terracidiphilus*

; f__

G12

-

WMSP1

_3

6

g__

PAUC26f; g__

*Chryseolinea*

;

c__Cyanobacteria

; c__

Betaproteobacteria

; g__

*Sphingomonas*

; g__

AMGG11

_4

2

f__

Sandaracinaceae

; g__

uncultured

-

Caulo

bacteraceae

_5

6

c__

UA11; g__

uncultured

-

Sand

aracinaceae; g__RB41; c__

Subgroup

17; c__

Subgroup

5; g__

11

-

24

_6

8

g__

*Dokdonella*

; f__

DA101

soil

group; g__

uncultured

-

Gemmat

imonadaceae; g__

*Rhodoplanes*

; g__

*Rhizobium*

;

g__uncultured

-

Bradyrhizobiaceae; o__Gaiellales; g__

*Arenimonas*

**B**

**Plant species**

**Number**

**of OTUs**

**OTUs**

*Betula*

*papyrifera*

*Alnus*

*rugosa*

*Picea*

sp.

93

g__

uncultured

-

Acidob

acteriaceae (Subgroup 1

); p__Parcubacteria; f__MSB

-

4B10; o__Ktedonobacterales;

g__uncultured

-

Simkaniaceae; p__Latescibacteria; g__

*Chthoniobacter*

; g__

*Aquicella*

; g__uncultured

-

Cytophagaceae; f__ODP1230B8.23; g__

*Pirellula*

; g__

*Haliangium*

; c__

Subgroup

6; o__X35; c__

Pla4

lineage; f__Chitinophagaceae; f__SM2D12; g__H16; g__

Pir4

lineage; g__

*Nocardioides*

; p__Proteobacteria;

o__

SAR324

clade(Marine group B

); g__

*Edaphobacter*

; g__

*Acidicapsa*

; g__uncultured

-

Planctomycetaceae;

o__Armatimonadales; c__Alphaproteobacteria; f__cvE6; f__

Xanthomonadales

*Incertae*

*sedis*;

g__

*Singulisphaera*

; g__

*Bryocella*

; p__

TM6

(Dependentiae

); g__

*Planctomyces*

; g__

*Candidatus*

*protochlamydia*; g*_*

_

*Gemmatimonas*

; g__

*Acidibacter*; f*_*

_DA111; f__

Acidobacteriaceae

(Subgroup 1

);

g__

*Candidatus*

*solibacter*; o*_*

Acidimicrobiales; c__BD7

-

11; f__Planctomycetaceae; p__Chloroflexi;

c__

OPB35

soil

group; f__Oligoflexaceae; g__

uncultured

*-*

*Chitinophagaceae*; f*_*

_Bradyrhizobiaceae;

g__

*Rhizomicrobium*

; c__Gammaproteobacteria; f__

Acidobacteriaceae (Subgroup 1

); g__

*Telmatobacter*

;

g__

*Legionella*

; p__Saccharibacteria; f__P3OB

-

42; g__

*Mucilaginibacter*

; g__

*Aciditerrimonas*

; c__OM190;

g__

*Phenylobacterium*

; f__Tepidisphaeraceae; g__

*Geobacter*

; g__

*Bdellovibrio*

; g__SM1A02; c__

KD4

-

96

c

__Candidatus

Azambacteria; c__vadinHA49; g__

*Neochlamydia*

; g__

*Gaiella*

; g__

*Anaeromyxobacter*

;

g__

*Bryobacter*

; g__

*Acidothermus*

; g__

*Coxiella*

; g__

*Burkholderia*

*-*

*Paraburkholderia; g_*

_

*Sorangium*

;

f__BIrii41; p__Omnitrophica; g__

*Flavobacterium*

; k__Bacteria; g__

*Acidobacterium*

; f__Planctomycetaceae;

f__

env.OPS

17; f__Fimbriimonadaceae; c__JG37

-

AG

-

4; c__

Subgroup

2; g__

*Opitutus*

; g__

*Granulicella*

;

c__TK10; o__HTA4; o__Ktedonobacterales; o__C0119; g__

*Gemmata*

; o__Obscuribacterales;

g__

*Jatrophihabitans*; f*_*

_

0319

-

6G20

*Betula*

*papyrifera*

*Picea*

sp.

3

g__

*Isosphaera*

; p__

Woesearchaeota

(DHVEG

-

6

); g__

AMGG11

*Alnus*

*rugosa*

*Picea*

sp.

3

f

__

Elev

-

16S

-

1332; f__mle1

-

27; g__

*Ferruginibacter*

*Betula*

*papyrifera*

4

g__

*Pseudomonas*

; p__FCPU426; g__

*Terracidiphilus*

; f__

G12

-

WMSP1

*Alnus*

*rugosa*

4

g__

*Candidatus*

*xiphinematobacter*

; g__

*Chryseolinea*

; g__

*Phaselicystis*

;

c__

Cyanobacteria

*Picea*

sp.

1

c__

Betaproteobacteria
