# Supplementary material for: Vegetation drives the structure of active microbial communities on an acidogenic mine tailings deposit
Source: PeerJ. 2020 Oct 21;8:e10109. doi: 10.7717/peerj.10109 (PMC7585372; doi:10.7717/peerj.10109)
Supplement: Supplemental Information 6 — Venn’s table showing shared and unique fungal OTUs, from the 100 most abundant taxa, between vegetation density classes (A) and plant species (B). The letter in front of the taxon represents the maximum depth of taxonomy (g: genus, f: family, c: class, o: order, p: phylum, k: kingdom). [file peerj-08-10109-s006.docx]

**Table S6.** Venn’s table showing shared and unique fungal OTUs, from the 100 most abundant taxa, between vegetation density classes (A) and plant species (B). The letter in front of the taxon represents the maximum depth of taxonomy (g: genus, f: family, c: class, o: order, p: phylum, k: kingdom).

**A**

**Vegetation density**

**classes**

**Number of**

**OTUs**

**OTUs**

_1 _2 _3 _4 _5 _6

62

g__

*Venturia*

; o__

Rhizophydiales

; g__

*Pateramyces*

; g__

*Tomentella*

; f*_*

_

Hyaloscyphaceae

;

g__

*Claussenomyces*

; g__

*Claroideoglomus*

; g__

*Archaeospora*

; g__

*Occultifur*

; g__

*Mortierella*

;

g__

*Cladophialophora*

; f__

Helotiaceae

; g__

*Blastocladiella*

; o__

Pezizales

;

p__Ascomycota

;

g__

*Cryptococcus*

(f__

Filobasidiaceae

); g__

*Cryptotrichosporon*

;

k__Fungi

; g__

*Hypocrea*

; g__

*Articulospora*

;

g__

*Prosthemium*

; g__

*Epicoccum*

; g__

*Thelephora*

; g__

*Pseudaegerita*

; g__

*Russula*

; g__

*Alatospora*

;

o__

Pleosporales

; o__

Helotiales

; g__

*Oidiodendron*

; g__

*Cistella*

; g__

*Capronia*

; g__

*Hyaloscypha*

;

c__

Leotiomycetes

; g__

*Tylospora*

; g__

*Meredithblackwellia*

; o__

Hypocreales

; c__

Sordariomycetes

;

c__

Incertae

sedis

; o__

Agaricales

; g__

*Eupenicillium*

; g__

*Schizangiella*

; g__

*Rhizoscyphus*

; g__

*Meloderma*

;

c__

Chytridiomycetes

; g__

*Dissophora*

; c__

Agaricomycetes

;

p__Basidiomycota

; g__

*Anthracoidea*

;

p__

Chytridiomycota

; g__

*Mollisia*

; f__

Pezizaceae

; g__

*Lactarius*

; g__

*Inocybe*

; g__

*Cortinarius*

;

c__

Eurotiomycetes

; g__

*Serendipita*

; g__

*Rhizophagus*

; c__

Lecanoromycetes

; g__

*Phialocephala*

;

g__

*Monoblepharis*

; g__

*Archaeorhizomyces*

; g__

*Amphinema*

_1 _2 _3 _4 _5

2

g__

*Gibberella*

; g__

*Paraglomus*

_1 _2 _3 _5 _6

1

g__

*Pandora*

_1 _2 _4 _5 _6

4

g__

*Clavaria*

; g__

*Thelebolus*

; f__

Tricholomataceae

; g__

*Exophiala*

_1 _2 _3 _4

1

g__

*Leuconeurospora*

_1 _2 _3 _6

3

g__

*Ceratobasidium*

; g__

*Devriesia*

; g__

*Parastagonospora*

_1 _2 _5 _6

2

g__

*Lecythophora*

; f__

Teratosphaeriaceae

_1 _3 _4 _5

1

f__

Herpotrichiellaceae

_1 _4 _5 _6

1

g__

*Cenococcum*

_2 _3 _4 _5

1

g__

*Hebeloma*

_2 _4 _5 _6

1

g__

*Mycoarthris*

_3 _4 _5 _6

1

g__

*Lambertella*

_1 _2 _3

2

g__

*Kurtzmanomyces*

; g__

*Neofabraea*

_1 _2 _4

4

g__

*Trichoderma*

; g__

*Laccaria*

; g__

*Acephala*

; g__

*Entrophospora*

_1 _2 _6

2

c__

Dothideomycetes

; g__

*Exobasidium*

_1 _3 _6

1

g__

*Schizosaccharomyces*

_1 _4 _6

1

g__

*Trechispora*

_1 _5 _6

1

g__

*Tothia*

_2 _3 _4

2

o__

Xylariales

; f__

Clavicipitaceae

_3 _4 _5

1

g__

*Wilcoxina*

_3 _5 _6

2

g__

*Glomus*

; g__

*Cudoniella*

_4 _5 _6

2

g__

*Pseudotomentella*

; g__

*Neonectria*

_1 _2

3

g__

*Microbotryozyma*

; o__

Capnodiales

; g__

*Arachnopeziza*

_1 _3

2

f__

Verrucariaceae

; c__

Glomeromycetes

_1 _4

1

f__Trichocomaceae

_1 _6

1

g__

*Mycena*

_2 _3

1

g__

*Trichopeziza*

_2 _4

3

g__

*Paxillus*

; g__

*Phlogicylindrium*

; g__

*Acaulospora*

_2 _5

1

g__

*Geopora*

_3 _4

2

g__

*Discostroma*

; g__

*Chloridium*

_3 _5

1

f__Nectriaceae

_4 _5

1

f__Amphisphaeriaceae

_4 _6

1

g__

*Alnicola*

_5 _6

4

g__

*Sebacina*

*; g_*

_

*Piloderma*

; g__

*Filobasidium*

; g__

*Pseudeurotium*

_1

5

g__

*Sclerotinia*

; f__

Parmeliaceae

; g__

*Penidiella*

; g__

*Acidomyces*

; g__

*Fusarium*

_2

5

g__

*Peziza*

; g__

*Gymnopilus*

; g__

*Rhizoctonia*

; g*_*

_

*Piriformospora*

; g__

*Taphrina*

_3

14

g__

*Lanzia*

; g__

*Podospora*

; g__

*Septoglomus*

; f__

Geoglossaceae

; g__

*Atla*

; g__

*Hygrocybe*

;

g__

*Metarhiziopsis*

; g__

*Toninia*

; g__

*Tricharina*

; g_

*_*

*Anthostomella*

; g__

*Trichosporon*

; g__

*Lophodermium*

;

g__

*Leptosphaeria*

; f__

Botryosphaeriaceae

_4

8

g__

*Clitopilus*

; g__

*Hyphodontia*

; f__

Sebacinales

Group

B; g__

*Mycovellosiella*

; f__

Sebacinaceae

;

g__

*Coccomyces*

; f__

Ceratobasidiaceae

; g__

*Tirmania*

_5

11

g__

*Kalaharituber*

; g__

*Phacidium*

; g__

*Umbelopsis*

; g__

*Tricladium*

; g__

*Tricholosporum*

; g__

*Neobulgaria*

;

g__

*Ramariopsis*

; g__

*Monacrosporium*

; f__

Pyronemataceae

; g__

*Rhinocladiella*

; g__

*Phaeomollisia*

_6

10

g__

*Apodus*

; g__

*Entoloma*

; g__

*Lycoperdon*

; c__

Microbotryomycetes

; g__

*Sympoventuria*

;

g__

*Leucosporidiella*

; g__

*Cylindrosympodium*

; g__

*Pseudogymnoascus*

; g__

*Chamaeleomyces*

; g__

*Mrakia*

**B**

**Plant species**

**Number of**

**OTUs**

**OTUs**

*Betula*

*papyrifera*

*Alnus*

*rugosa*

*Picea*

sp.

69

g__

*Venturia*

; o__

Rhizophydiales

; g__

*Pateramyces*

; g__

*Tomentella*

; g__

*Trichoderma*

;

f__

Hyaloscyphaceae

; g__

*Claussenomyces*

; g__

*Claroideoglomus*

; g__

*Archaeospora*

; g__

*Occultifur*

;

g__

*Mortierella*

; g__

*Cladophialophora*

; g__

*Blastocladiella*

; g__

*Gibberella*

; g__

*Clavaria*

; o__

Pezizales

;

p__Ascomycota

;

g__

*Cryptococcus*

(f__

Filobasidiaceae

); g__

*Cryptotrichosporon*

;

k__Fungi

; g__

*Hypocrea*

;

f__

Tricholomataceae

; g__

*Articulospora*

; g__

*Prosthemium*

; g__

*Epicoccum*

; g__

*Thelephora*

;

g__

*Pseudaegerita*

; g__

*Russula*

; g__

*Alatospora*

; o__

Pleosporales

; o__

Helotiales

; g__

*Oidiodendron*

;

g__

*Cistella*

; g__

*Capronia*

; f*_*

_

Clavicipitaceae

; g__

*Hyaloscypha*

; c__

Leotiomycetes

; g__

*Tylospora*

;

g__

*Meredithblackwellia*

; o__

Hypocreales

; g__

*Wilcoxina*

; c__

Sordariomycetes

; c__

Incertae

sedis

;

o__

Agaricales

; g__

*Eupenicillium*

; g__

*Schizangiella*

; f__

Herpotrichiellaceae

; g__

*Rhizoscyphus*

;

g__

*Meloderma*

; c__

Chytridiomycetes

; g__

*Dissophora*

; c__

Agaricomycetes

;

p__Basidiomycota

;

g__

*Anthracoidea*

; p__

Chytridiomycota

; g__

*Mollisia*

; f__

Pezizaceae

; g__

*Inocybe*

; g__

*Lactarius*

;

g__

*Cortinarius*

; c__

Eurotiomycetes

; g__

*Serendipita*

; g__

*Mycoarthris*

; g__

*Rhizophagus*

;

c__

Lecanoromycetes

; g__

*Phialocephala*

; g__

*Monoblepharis*

; g__

*Archaeorhizomyces*

; g__

*Amphinema*

*Betula*

*papyrifera*

*Alnus*

*rugosa*

4

o__

Xylariales

; g__

*Phlogicylindrium*

; g__

*Hebeloma*

; g__

*Arachnopeziza*

*Betula*

*papyrifera*

*Picea*

sp.

9

f__

Helotiaceae

; g__

*Septoglomus*

; g__

*Acephala*

; g__

*Neofabraea*

; g__

*Devriesia*

; g__

*Hygrocybe*

;

g__

*Leuconeurospora*

; g__

*Cudoniella*

; f__

Botryosphaeriaceae

*Alnus*

*rugosa*

*Picea*

sp.

10

g__

*Lambertella*

; g__

*Thelebolus*

; g__

*Ceratobasidium*

; f__

Sebacinales

Group

B; g__

*Paraglomus*

;

g__

*Chloridium*

; g__

*Anthostomella*

; g__

*Acaulospora*

; g__

*Leptosphaeria*

; g__

*Neonectria*

*Betula*

*papyrifera*

18

g__

*Gymnopilus*

; g__

*Clitopilus*

; g__

*Laccaria*

; g__

*Lecythophora*

; g__

*Hyphodontia*

; f__

Geoglossaceae

;

g__

*Paxillus*

; g__

*Trechispora*

; f__

Verrucariaceae

;

g__

*Glomus*

; g__

*Toninia*

; f*_*

_

Sebacinaceae

;

g__

*Pseudotomentella*

; g__

*Cenococcum*

; g*_*

_

*Parastagonospora*

; f__

Amphisphaeriaceae

; f__

Nectriaceae

;

f__

Trichocomaceae

*Alnus*

*rugosa*

17

g__

*Apodus*

; g__

*Peziza*

; g__

*Geopora*

; c__

Dothideomycetes

; g__

*Alnicola*

; f__

Xylariaceae

;

g__

*Entrophospora*

; g__

*Discostroma*

; g__

*Filobasidium*

; g__

*Rhizoctonia*

; g__

*Piriformospora*

;

g__

*Exophiala*

;

g__

*Pandora*

; c__

Glomeromycetes

; g__

*Tricharina*

; g__

*Dactylellina*

; f__

Ceratobasidiaceae

*Picea*

sp.

12

g__

*Podospora*

; g__

*Schizosaccharomyces*

; g__

*Metarhiziopsis*

; g__

*Mycovellosiella*

; g__

*Coccomyces*

;

g__

*Trichosporon*

; g__

*Lophodermium*

; g__

*Trichopeziza*

; g__

*Davidiella*

; g__

*Tirmania*

;

f__

Teratosphaeriaceae

; g__

*Taphrina*
