# Supplementary material for: Vegetation drives the structure of active microbial communities on an acidogenic mine tailings deposit
Source: PeerJ. 2020 Oct 21;8:e10109. doi: 10.7717/peerj.10109 (PMC7585372; doi:10.7717/peerj.10109)
Supplement: Supplemental Information 7 — SD: standard-deviation, VDC: vegetation density classes, p_s: plant species. [file peerj-08-10109-s007.docx]

**Table S7.** Relative abundance of transcripts (%) of the 6 most abundant bacteria (A) and fungi (B) classes between the different vegetation density classes and plant species. SD: standard-deviation, VDC: vegetation density classes, p_s: plant species.

**A**

| VDC or p_s | | Gamma- proteobacteria | Delta-proteobacteria | Alpha-proteobacteria | Plancto-mycetacia | Acido-bacteria | Actino-bacteria |
| --- | --- | --- | --- | --- | --- | --- | --- |
| VDC-1 | Relative abundance (%) | 9.88 | 10.00 | 8.29 | 8.22 | 6.43 | 2.84 |
|  | SD | 2.29 | 2.50 | 1.23 | 1.34 | 3.64 | 0.41 |
| VDC-2 | Relative abundance (%) | 11.46 | 9.69 | 8.89 | 7.60 | 5.80 | 4.09 |
|  | SD | 6.70 | 2.32 | 2.20 | 1.38 | 3.73 | 5.04 |
| VDC-3 | Relative abundance (%) | 10.23 | 10.82 | 8.98 | 8.57 | 4.26 | 3.34 |
|  | SD | 6.19 | 2.40 | 1.16 | 1.71 | 1.99 | 0.88 |
| VDC-4 | Relative abundance (%) | 10.23 | 10.04 | 9.78 | 7.72 | 5.12 | 3.77 |
|  | SD | 6.34 | 2.43 | 3.26 | 1.50 | 2.77 | 2.66 |
| VDC-5 | Relative abundance (%) | 7.86 | 10.30 | 8.72 | 9.28 | 4.11 | 3.06 |
|  | SD | 1.86 | 1.17 | 1.01 | 1.67 | 1.06 | 0.88 |
| VDC-6 | Relative abundance (%) | 8.40 | 10.80 | 10.18 | 6.95 | 3.88 | 5.67 |
|  | SD | 1.97 | 1.21 | 1.09 | 1.02 | 0.78 | 2.40 |
| *Alnus rugosa* | Relative abundance (%) | 9.72 | 10.36 | 10.37 | 7.42 | 4.61 | 4.68 |
|  | SD | 5.58 | 2.87 | 3.77 | 1.34 | 2.37 | 6.50 |
| *Betula papyrifera* | Relative abundance (%) | 11.39 | 9.52 | 8.45 | 8.19 | 5.50 | 3.30 |
|  | SD | 6.93 | 1.73 | 1.56 | 1.72 | 3.58 | 1.28 |
| *Picea* sp. | Relative abundance (%) | 9.76 | 10.56 | 9.29 | 7.80 | 5.41 | 3.74 |
|  | SD | 6.54 | 2.67 | 1.58 | 1.36 | 3.04 | 1.50 |

**B**

| VDC or p_s | | Others (with no phylum) | Agaricomycetes | Leotiomycetes | Others (with Ascomycota phyla) | *Incertae sedis* | Sordario-mycetes |
| --- | --- | --- | --- | --- | --- | --- | --- |
| VDC-1 | Relative abundance (%) | 30.39 | 12.29 | 18.64 | 14.51 | 3.57 | 2.30 |
|  | SD | 13.24 | 9.23 | 15.0 | 8.24 | 1.43 | 0.86 |
| VDC-2 | Relative abundance (%) | 27.04 | 19.10 | 18.65 | 11.32 | 4.40 | 3.63 |
|  | SD | 8.60 | 9.23 | 13.26 | 6.73 | 2.68 | 3.32 |
| VDC-3 | Relative abundance (%) | 27.72 | 26.34 | 8.40 | 9.82 | 4.46 | 8.34 |
|  | SD | 8.54 | 11.27 | 8.10 | 4.17 | 1.58 | 6.31 |
| VDC-4 | Relative abundance (%) | 26.75 | 22.87 | 14.21 | 9.45 | 5.52 | 4.50 |
|  | SD | 7.55 | 11.16 | 11.62 | 3.01 | 4.78 | 3.61 |
| VDC-5 | Relative abundance (%) | 26.91 | 20.31 | 5.97 | 12.82 | 12.74 | 4.18 |
|  | SD | 6.89 | 13.48 | 3.42 | 7.99 | 13.29 | 2.69 |
| VDC-6 | Relative abundance (%) | 27.21 | 11.29 | 5.70 | 9.62 | 19.66 | 4.64 |
|  | SD | 7.38 | 3.89 | 12.15 | 2.33 | 11.59 | 2.06 |
| *Alnus rugosa* | Relative abundance (%) | 25.51 | 23.51 | 13.57 | 9.25 | 4.89 | 6.36 |
|  | SD | 6.92 | 10.43 | 10.83 | 2.82 | 3.21 | 5.54 |
| *Betula papyrifera* | Relative abundance (%) | 27.00 | 21.23 | 17.88 | 10.21 | 4.65 | 3.63 |
|  | SD | 8.66 | 11.24 | 13.15 | 4.61 | 3.03 | 2.52 |
| *Picea* sp. | Relative abundance (%) | 28.36 | 21.17 | 12.95 | 10.99 | 5.64 | 4.20 |
|  | SD | 7.93 | 10.24 | 11.79 | 6.43 | 5.39 | 2.57 |
